# CRISPR/Pepper‐tDeg: A Live Imaging System Enables Non‐Repetitive Genomic Locus Analysis with One Single‐Guide RNA

**DOI:** 10.1002/advs.202402534

**Published:** 2024-06-26

**Authors:** Meng Chen, Xing Huang, Yakun Shi, Wen Wang, Zhan Huang, Yanli Tong, Xiaoyong Zou, Yuzhi Xu, Zong Dai

**Affiliations:** ^1^ Guangdong Provincial Key Laboratory of Sensing Technology and Biomedical Instrument School of Biomedical Engineering Shenzhen Campus of Sun Yat‐Sen University Sun Yat‐Sen University Shenzhen 518107 China; ^2^ School of Pharmaceutical Sciences Sun Yat‐Sen University Guangzhou 510275 China; ^3^ School of Chemistry Sun Yat‐Sen University Guangzhou 510275 China; ^4^ Scientific Research Center The Seventh Affiliated Hospital Sun Yat‐Sen University Shenzhen 518107 China

**Keywords:** CRISPR‐Cas9, fluorogenic protein, genomic loci labeling, live cell imaging, non‐repetitive sequences

## Abstract

CRISPR‐based genomic‐imaging systems have been utilized for spatiotemporal imaging of the repetitive genomic loci in living cells, but they are still challenged by limited signal‐to‐noise ratio (SNR) at a non‐repetitive genomic locus. Here, an efficient genomic‐imaging system is proposed, termed CRISPR/Pepper‐tDeg, by engineering the CRISPR sgRNA scaffolds with the degron‐binding Pepper aptamers for binding fluorogenic proteins fused with Tat peptide derived degron domain (tDeg). The target‐dependent stability switches of both sgRNA and fluorogenic protein allow this system to image repetitive telomeres sensitively with a 5‐fold higher SNR than conventional CRISPR/MS2‐MCP system using “always‐on” fluorescent protein tag. Subsequently, CRISPR/Pepper‐tDeg is applied to simultaneously label and track two different genomic loci, telomeres and centromeres, in living cells by combining two systems. Given a further improved SNR by the split fluorescent protein design, CRISPR/Pepper‐tDeg system is extended to non‐repetitive sequence imaging using only one sgRNA with two aptamer insertions. Neither complex sgRNA design nor difficult plasmid construction is required, greatly reducing the technical barriers to define spatiotemporal organization and dynamics of both repetitive and non‐repetitive genomic loci in living cells, and thus demonstrating the large application potential of this genomic‐imaging system in biological research, clinical diagnosis and therapy.

## Graphical abstract

The live CRISPR/Pepper‐tDeg imaging system, as one of the few successful examples for visualizing non‐repetitive genomic loci by only one sgRNA, utilizes fluorogenic split protein design to significantly improve signal‐to‐noise ratio (SNR). It has such distinct features as minimalist design, easy operation, and comparable accuracy with gold standard FISH, which is promising in biological research, clinical diagnosis and therapeutic evaluation.

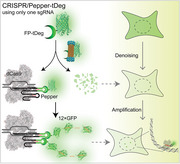

## 1 Introduction

Live‐cell imaging of non‐repetitive genomic loci, which are the great majority of the human genome, is highly helpful to the thorough understanding of the genome function and mechanism related to its organization and dynamics, including gene transposition, deletion, duplication, translocation and interactions with RNA and proteins.^[^ ^1^, ^2^, ^3^, ^4^, ^5^ ^]^ In recent years, the clustered regularly interspaced short palindromic repeats (CRISPR)‐CRISPR‐associated protein (Cas) systems using nuclease‐deactivated Cas9 (dCas9) have been applied for imaging repetitive genomic loci of interest with high precision and flexibility by changing the single‐guide RNA (sgRNA) spacer with a short 20‐nucleotide sequence.^[^ ^6^, ^7^, ^8^, ^9^, ^10^, ^11^, ^12^, ^13^, ^14^, ^15^, ^16^, ^17^, ^18^, ^19^, ^20^, ^21^, ^22^ ^]^ However, the relatively low signal‐to‐noise ratio (SNR) especially at a non‐repetitive genomic locus, in which there is only one copy of anchored dCas9‐sgRNA complex emitting insufficient fluorescence signal, is a primary challenge to overcome.

Typically, to amplify the signal and achieve an enhanced SNR, a common way is to modify dCas9^[^ ^8^ ^]^ or sgRNA^[^ ^14^ ^]^ to carry multiple copies of fluorescent proteins (FPs), but extensive modification may decrease each component's expression and stability, thus decreasing the labeling efficiency.^[^ ^17^ ^]^ To reduce the impact on stability, two approaches, including CRISPR‐Sirius with an optimized sgRNA carrying an octet array of aptamers (MS2 or PP7),^[^ ^16^ ^]^ and Suntag tethering dCas9 with tandem repeats of peptide array,^[^ ^23^, ^24^ ^]^ were integrated in the dCas9‐sgRNA system. However, the SNR is still insufficient for the non‐repetitive locus labeling just by amplifying the signals, as the constitutively fluorescent tags emit “always‐on” signal with high background noise. To reduce the background noise, several techniques were proposed, including bimolecular fluorescence complementation (BiFC),^[^ ^25^, ^26^ ^]^ LiveFISH,^[^ ^18^ ^]^ and CRISPR/MB hybrid system.^[^ ^17^, ^27^ ^]^ The BiFC system used the split fragments of fluorescent proteins that can reconstitute to form intact fluorescent proteins only when each non‐fluorescent fragment was recruited to the same locus,^[^ ^26^ ^]^ while the LiveFISH system also possessed reduced background noise through the fast degradation of unbound Cyanine 3‐labeled sgRNA (Cy3‐sgRNA).^[^ ^18^ ^]^ The CRISPR/MB hybrid system modified sgRNA to carry molecular beacons (MB)^[^ ^17^, ^27^ ^]^ to obtain an excellent SNR, which successfully imaged non‐repetitive genomic loci with several sgRNAs. However, LiveFISH and CRISPR/MB required to deliver exogenous ribonucleoproteins and nucleic acids to live cells, respectively, rendering the retention time sub‐optimal. Moreover, current labeling approaches for non‐repetitive sequences usually required dozens of different sgRNAs delivered simultaneously per target locus to achieve sufficient signal amplification, resulting in higher risk of off‐target sites by multiple sgRNAs.^[^ ^28^ ^]^

As labeling non‐repetitive sequences using only one sgRNA is generally inefficient and remains a huge challenge, only few latest techniques have made this effort,^[^ ^29^, ^30^, ^31^ ^]^ but there are such technical difficulties as extensive sgRNA modifications or complex plasmid constructions, as well as the high background noise induced from the constitutively fluorescent tags, precluding the broad applications. Alternative strategies to decrease the fluorescence background and improve the SNR were involved by inserting fluorogenic RNA aptamers (e.g, broccoli) into sgRNA,^[^ ^16^, ^19^, ^32^ ^]^ or using RNA aptamer‐stabilized fluorogenic protein,^[^ ^45^ ^]^ but the contrast of fluorogenic aptamer/protein is yet insufficient for non‐repetitive sequence analysis. Therefore, a new efficient and facile method for non‐repetitive sequence imaging with sufficiently high SNR is still highly desirable.

In the fluorogenic protein technology developed recently,^[^ ^34^ ^]^ “tDeg”, a Tat peptide derived degron domain which contains a degron sequence and a Tat peptide, was fused to the C‐terminus of fluorescent proteins (FP‐tDeg). Due to the degron sequence, FP‐tDeg can be recognized and rapidly degraded by the ubiquitin‐proteasome system (“turn‐off”), unless it binds to the Tat peptide‐binding RNA aptamer “Pepper”, which shields the degron and thus prevents FP‐tDeg from degradation, generating an activated fluorescent signal (“turn‐on”).^[^ ^35^ ^]^ Integrating Pepper aptamers and FP‐tDeg in the CRISPR‐dCas9 system can reduce the background fluorescence as all unbound fluorogenic proteins were degraded.^[^ ^33^ ^]^ But for non‐repetitive sequence imaging, we put forward two key factors, which make CRISPR‐dCas9 system simple but powerful for labeling non‐repetitive genomic loci: (1) The appropriate expression level of the degradable fluorogenic protein in nucleus—too little and the target labeling is insufficient, while too much would exceed the degradation rate and result in a high background signal; (2) Robust signal amplification manner without noise magnification, which can be achieved by integrating tandem split fluorescent protein design to the degradable fluorogenic protein; however, when only tandem split GFP tag was available, background noise distracted the non‐repetitive sequence imaging so much that multiplex sgRNAs were still required.^[^ ^36^ ^]^

Herein, based on the abovementioned two key factors, we developed a robust and efficient genomic‐imaging system, termed CRISPR/Pepper‐tDeg, using the target‐induced stabilization of fluorogenic CRISPR−protein complexes for accurate quantification of repetitive and non‐repetitive genomic loci, as well as for dual‐color tracking of chromatin dynamics in living cells. This system relies on one sgRNA engineered with the degron‐binding Pepper aptamers in the stem loops, and a fusion protein with a C‐terminal degron, an N‐terminal nuclear localization signal (NLS), and a tandem of fluorescent proteins under the control of UbC promoter to drive appropriate fluorogenic protein expression in nucleus. The target‐dependent stability switches of sgRNAs and fluorogenic proteins enable the CRISPR/Pepper‐tDeg system to sensitively label genomic loci with a 5‐fold higher SNR than the conventional genomic‐imaging system CRISPR/MS2‐MCP that depends on the constitutively fluorescent protein tag emitting “always‐on” signal.^[^ ^10^, ^11^, ^12^ ^]^ By combination with CRISPR/MS2‐MCP, CRISPR/Pepper‐tDeg system allows to simultaneously visualize and track the real‐time movement of repetitive telomeres and centromeres in living cells. Moreover, by coupled with the split fluorescent protein design, the split‐GFP‐coupled CRISPR/Pepper‐tDeg system was further extended to afford sufficient SNR for non‐repetitive sequence imaging using only one sgRNA with two aptamer insertions. The CRISPR/Pepper‐tDeg system shows great promise as a convenient but powerful tool for genomic imaging in applications such as biomedical research and clinical therapy.

## 2 Results and Discussion

### 2.1 Design and Fabrication of CRISPR/Pepper‐tDeg System

**Scheme 1:**
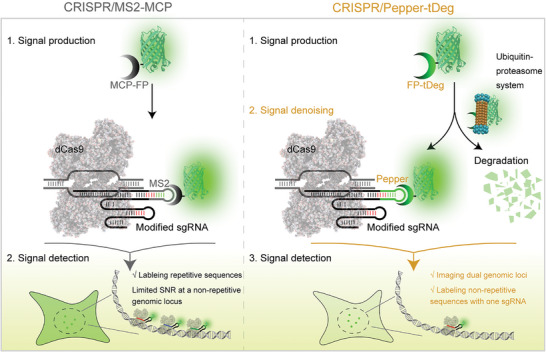
Principle for live‐cell imaging of genomic loci by conventional CRISPR/MS2‐MCP system (left) and CRISPR/Pepper‐tDeg system (right). In the conventional CRISPR/MS2‐MCP system (left), the MCP‐fused fluorescent proteins have excessive expression (≈million copies) and emit strong “always‐on” signal even in unbound form, resulting in a high background. Suffering from this low SNR, the minimum fluorescent proteins that are bound to the non‐repetitive genomic loci with only one copy of anchored dCas9‐sgRNA complex, cannot be distinguished from the unbound fluorescent proteins. Thus, CRISPR/MS2‐MCP fails to be used for imaging non‐repetitive genomic loci, unless dozens of different sgRNAs are simultaneously delivered per target locus in the same cell to achieve sufficient signal amplification. Conversely, in our CRISPR/Pepper‐tDeg system (right), the fast degradation of tDeg‐fused fluorescent proteins (FP‐tDeg) in unbound form by the ubiquitin‐proteasome system guarantees the low background with “turn‐off” signal, unless they bind to the Pepper‐containing sgRNA in the target‐bound CRISPR complex, switching on the fluorescence. Therefore, with a simple signal amplification technique using tDeg‐fused split fluorescent proteins, CRISPR/Pepper‐tDeg system can obtain a much‐improved SNR, enabling robust imaging of a non‐repetitive genomic locus using only one sgRNA with two Pepper insertions.

To label and visualize specific non‐repetitive genomic loci with high efficiency, a CRISPR/Pepper‐tDeg system has been developed, which comprises an engineered sgRNA carrying two Pepper stem loops and a fluorescent protein reporter fused with tDeg (**Scheme** **1**, right). Taking advantage of the target DNA‐induced stabilization of fluorogenic CRISPR−protein complexes, this system was designed to possess a high SNR based on two key thoughts: (1) dual signal denoising mediated by the rapid degradation of both unbound sgRNA and unbound fluorogenic proteins, and simultaneously, (2) dual signal amplification by a modest number of Pepper aptamers inserted in the stem loops of sgRNA, and a FP‐tDeg fusion tagged with a tandem of fluorescent proteins. In this design, only when bound to target DNA can CRISPR trigger cascade stabilization of sgRNA and fluorogenic proteins, protecting them from RNase and protease degradation, respectively. In this way, unbound sgRNA is rapidly degraded,^[^ ^18^ ^]^ resulting in the subsequent rapid degradation of unbound tDeg‐fused fluorescent protein reporter with a low fluorescence background. In contrast, target‐bound fluorogenic CRISPR−protein complexes remain stable due to the shielded sgRNA and the shielded degron of the fusion proteins, which prevent recognition of RNase and the ubiquitin‐proteasome system from degradation, respectively, generating activated fluorescent signals for genomic imaging. Unlike the conventional CRISPR/MS2‐MCP system, which are still afflicted by the high background signal from the constitutively fluorescent (“always‐on”) proteins when imaging non‐repetitive sequences (Scheme 1, left), there is no need to deliver multiplex sgRNAs or integrate dozens of RNA aptamers into one sgRNA to achieve distinct target signal from background. Instead, sensitive imaging of non‐repetitive sequences is achieved by integrating the CRISPR/Pepper‐tDeg system with the split fluorescent protein design and one sgRNA engineered with only two Pepper aptamers. Therefore, this target‐dependent stability switch of fluorogenic proteins enables CRISPR/Pepper‐tDeg system to accurately detect repetitive and even non‐repetitive sequences. By further combination with current genomic‐labeling methods like CRISPR/MS2‐MCP, CRISPR/Pepper‐tDeg system can visualize and real‐time track the movement of telomeres and centromeres in living cells simultaneously, showing the high complementarity with each other.

**Figure 1:**
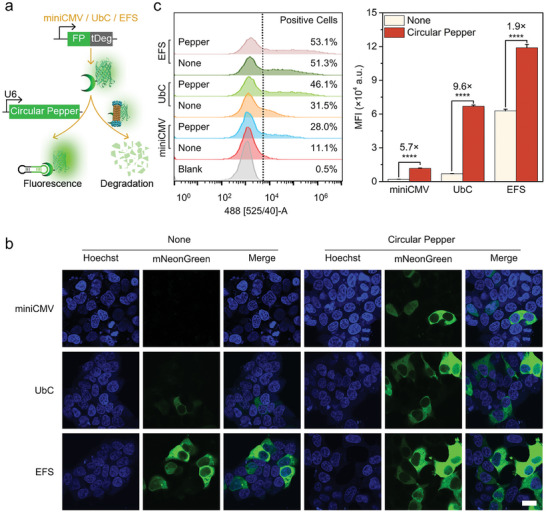
Promoter optimization using Pepper‐tDeg system. a) Schematic diagram of Pepper‐tDeg system supported by a circular Pepper aptamer. b) Confocal images for HEK293T cells expressing (mNeonGreen)_4_‐tDeg under the control of miniCMV, UbC, and EFS promoters with none or circular Pepper. Scale bar: 20 µm. c) Flow cytometry profiles and the mean fluorescence intensity (MFI) of corresponding cells. Data are means ± SD, *n* = 3. ****p < 0.0001 using two‐tailed *t*‐test.

First the CRISPR/Pepper‐tDeg system was examined in HEK293T cells for labeling repetitive telomeres with one telomere‐targeted sgRNA (sgTelo‐Pepper), which showed less fine labeling of genomic loci (Figure S1, sequences shown in Table S1, Supporting Information). Thus, two important parameters including the option of the promoter and the introduction of NLS sequences to drive appropriate expression of FP‐tDeg in the nucleus were optimized. Three promoters including miniCMV,^[^ ^34^ ^]^ UbC,^[^ ^34^ ^]^ and EFS^[^ ^37^ ^]^ were used to drive the expression of mNeonGreen‐tDeg, and compared in non‐Pepper‐ and circular‐Pepper‐expressing cells using the Tornado expression system,^[^ ^38^ ^]^ a circularization system assisted by ribozyme to make mammalian cells express RNA aptamer highly and stably (**Figure** **1**a). After transfection of Tornado plasmids (see Table S2, Supporting Information for detailed plasmid information), the successful and similar expression of circular Pepper among the three different promoter‐driven mNeonGreen‐tDeg‐expressing groups was confirmed by qRT‐PCR (Figure S2, Supporting Information). Without expressing Pepper, HEK293T cells showed negligible fluorescence of mNeonGreen when using the miniCMV and UbC promoters (Figure 1b). The fluorescence of mNeonGreen‐tDeg was decreased by 90% compared to that of unfused mNeonGreen, and was recovered by 43% through pretreatment with the proteasome inhibitor MG132 (Figure S3, Supporting Information), which indicated that the unbound mNeonGreen‐tDeg was degraded through the proteasomal pathway with high efficiency.^[^ ^39^ ^]^ In contrast, the fluorescence of mNeonGreen increased in circular‐Pepper‐expressing cells, especially with use of the UbC promoter. Notably, strong fluorescence of mNeonGreen was detected in either non‐Pepper‐ or circular‐Pepper‐expressing cells when using the EFS promoter. Additional qRT‐PCR confirmed the highest expression level of mNeonGreen‐tDeg driven by EFS promoter, which was 13.1 and 4.1 times as high as that driven by miniCMV and UbC promoter, respectively (Figure S4, Supporting Information). The relatively high expression of mNeonGreen‐tDeg driven by EFS promoter suggests that the expression of mNeonGreen‐tDeg may exceed its degradation, resulting in a strong fluorescence of mNeonGreen detected in either non‐Pepper‐ or circular‐Pepper‐expressing cells when using EFS promoter. Further confirmed by flow cytometry analysis, circular‐Pepper‐expressing cells exhibited a 5.7‐, 9.6‐, and 1.9‐fold increase in fluorescence relative to non‐Pepper‐expressing ones when using the miniCMV, UbC, and EFS promoters, respectively (Figure 1c). These results suggested that UbC was the optimal promoter for the appropriate expression of FP‐tDeg, which was used for further investigation.

**Figure 2:**
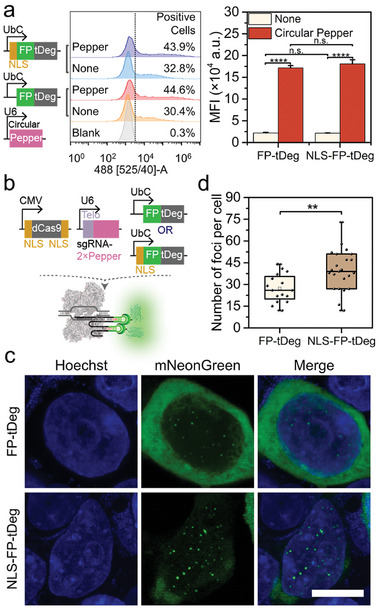
Optimization of FP‐tDeg nuclear localization. a) Flow cytometry profiles and the mean fluorescence intensity (MFI) for HEK293T cells expressing mNeonGreen‐tDeg and NLS‐mNeonGreen‐tDeg in Pepper‐tDeg system. Data are means ± SD, *n* = 3. b) Schematic diagram of CRISPR/Pepper‐tDeg system for genomic imaging using dCas9 fused with two NLSs, sgTelo‐Pepper and mNeonGreen‐tDeg with/without NLS insertion. c) Labeling of telomeres by CRISPR/Pepper‐tDeg system using mNeonGreen‐tDeg and NLS‐mNeonGreen‐tDeg. Images are maximum z projections. Scale bar: 10 µm. d) Box plot showing the total number of telomere foci detected per cell using mNeonGreen‐tDeg and NLS‐mNeonGreen‐tDeg; n  =  16 cells (left) and 23 cells (right). ****p < 0.0001, **p < 0.01, n.s., not significant using two‐tailed *t*‐test.

To improve the targeting efficiency, we made the first attempt to add NLS to the FP‐tDeg fusion, in addition to using dCas9 with two copies of NLS at the N‐terminus and C‐terminus respectively.^[^ ^11^ ^]^ It was reported that the fluorescent protein tag with an N‐terminal NLS was sterically better accessible to the genomic loci,^[^ ^6^ ^]^ hence we tested the FP‐tDeg fusion with an N‐terminal NLS (NLS‐FP‐tDeg) under the control of UbC promoter firstly in Pepper‐tDeg system (**Figure** **2**a). Similar to the FP‐tDeg fusion, the NLS‐FP‐tDeg fusion led to a significant fluorescence increase in circular‐Pepper‐expressing cells, suggesting that an N‐terminal NLS fusion did not alter the RNA‐dependent protein stabilization of FP‐tDeg (Figure 2a), ascribed to the nuclear ubiquitin‐proteasome system.^[^ ^40^ ^]^ Next, we tested the FP‐tDeg and NLS‐FP‐tDeg fusion in CRISPR/Pepper‐tDeg system and compared their labeling efficiency for repetitive telomeres (Figure 2b). The NLS‐FP‐tDeg fusion labeled the telomeres effectively, showing increased fluorescent foci observed (Figure 2c, down). In contrast, FP‐tDeg fusion without NLS exhibited only a few foci and decreased targeting specificity as the fluorescence substantially dispersed in cytoplasm (Figure 2c, top), and much lower labeling efficiency was obtained with FP‐tDeg fusion under the control of miniCMV promoter, which had only nucleolar signal observed (Figure S1, Supporting Information). After the insertion of NLS, the average number of telomeres per cell was increased by 1.5‐ fold (Figure 2d). These observations suggest that an improved labeling efficiency of CRISPR/Pepper‐tDeg system can be obtained using FP‐tDeg with the UbC promoter and NLS insertion. Next, the stability of optimized CRISPR/Pepper‐tDeg system was tested by time‐dependent imaging analysis after the stable HEK293T/dCas9 cells were transiently transfected with two plasmids expressing sgTelo‐Pepper and mNeonGreen‐tDeg in different time period. The time‐dependent imaging revealed that the mNeonGreen fluorescence foci can be clearly observed at 12 h post‐transfection and remained almost unchanged throughout 72 h, indicating that CRISPR/Pepper‐tDeg system has high stability in living cells (Figure S5, Supporting Information). Collectively, the UbC promoter and NLS insertion for FP‐tDeg, as well as over 12 h of transfection time are considered as optimal labeling conditions for subsequent imaging experiments.

### 2.2 Labeling Repetitive Genomic Loci Using CRISPR/Pepper‐tDeg

**Figure 3:**
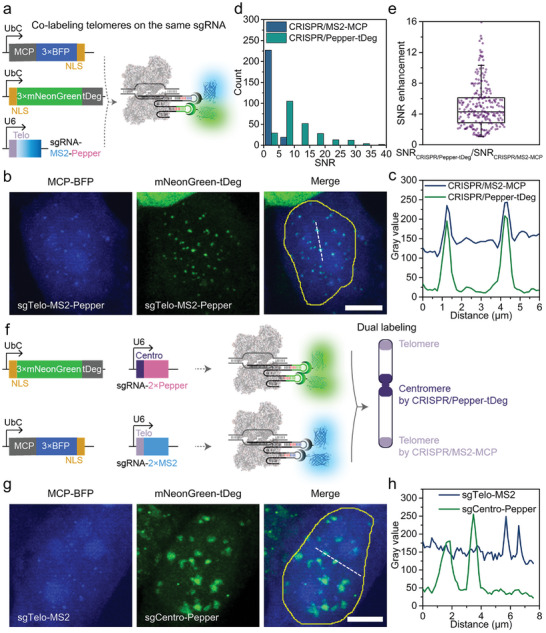
Dual‐color imaging of repetitive genomic loci using CRISPR/Pepper‐tDeg. a) Schematic diagram showing co‐labeling of telomeres by CRISPR/MS2‐MCP and CRISPR/Pepper‐tDeg using one sgTelo‐MS2‐Pepper. b) Co‐labeling of telomeres by CRISPR/MS2‐MCP (represented with MCP‐BFP) and CRISPR/Pepper‐tDeg (represented with mNeonGreen‐tDeg) using sgTelo‐MS2‐Pepper. Images are maximum z projections. Scale bar: 5 µm. c) The gray value profiles from CRISPR/MS2‐MCP (blue) and CRISPR/Pepper‐tDeg (green) along the dash line in (b). d) Histogram showing the SNR distribution of CRISPR/MS2‐MCP and CRISPR/Pepper‐tDeg for co‐labeling telomeres. 245 telomeres were analyzed from at least 15 cells. e) Box plot of ratios between SNR of CRISPR/Pepper‐tDeg and CRISPR/MS2‐MCP at each telomere locus. f) Schematic diagram showing simultaneous labeling of telomeres and centromeres by CRISPR/MS2‐MCP and CRISPR/Pepper‐tDeg using sgTelo‐MS2 and sgCentro‐Pepper. g) Simultaneous labeling of telomeres and centromeres by CRISPR/MS2‐MCP (represented with MCP‐BFP) and CRISPR/Pepper‐tDeg (represented with mNeonGreen‐tDeg) using sgTelo‐MS2 and sgCentro‐Pepper, respectively. Images are maximum z projections. Scale bar: 5 µm. h) The gray value profiles from sgTelo‐MS2 and sgCentro‐Pepper along the dash line in g).

To directly and homogeneously compare CRISPR/Pepper‐tDeg with conventional CRISPR/MS2‐MCP, we also made the first attempt to adopt the sgRNA fused with two aptamers, MS2 and Pepper, in stem loops (termed sgTelo‐MS2‐Pepper), as well as MCP‐BFP and mNeonGreen‐tDeg co‐transfected in the same cells (**Figure** **3**a). This integration allowed telomeres to be co‐labeled by CRISPR/MS2‐MCP (represented with MCP‐BFP signal) and CRISPR/Pepper‐tDeg (represented with mNeonGreen‐tDeg signal) simultaneously on the same sgRNA in the same cells without cell heterogeneity. Figure 3b,c showed that the identified puncta by MCP‐BFP and mNeonGreen‐tDeg were well co‐localized in the same cells. Similarly, the telomere foci were also found to be labeled by dual color when adopting two telomere‐targeted sgRNAs, one with MS2‐MS2 loops (called sgTelo‐MS2) and the other with Pepper‐Pepper loops (called sgTelo‐Pepper) in the same cells (Figure S6, Supporting Information). Next, we counted the SNR for telomeres co‐labelled by CRISPR/Pepper‐tDeg and CRISPR/MS2‐MCP. The histogram showed that conventional CRISPR/MS2‐MCP labeled 93% of telomeres with SNR < 5, while CRISPR/Pepper‐tDeg labeled 88% of telomeres with SNR > 5 (Figure 3d). It was worth noting that in contrast to the widely used CRISPR/MS2‐MCP system with relatively high background signal, the proposed CRISPR/Pepper‐tDeg system showed a better SNR, which was 5 times that of CRISPR/MS2‐MCP system (Figure 3e). We next examined whether CRISPR/Pepper‐tDeg system can be applied to label genomic loci in other human cell lines. Two human cell lines, human cervical carcinoma Hela cells and human osteosarcoma U2OS cells, with stable expression of dCas9 were constructed and expressed with CRISPR/Pepper‐tDeg and conventional CRISPR/MS2‐MCP targeting telomeres by using sgTelo‐Pepper and sgTelo‐MS2, respectively. Similar with those results obtained in HEK293T cells, CRISPR/Pepper‐tDeg system showed clear telomere foci with relatively low background signal, which had a higher SNR than conventional CRISPR/MS2‐MCP system in both Hela and U2OS cells (Figure S7, Supporting Information), indicating that CRISPR/Pepper‐tDeg enables the efficient labeling of genomic loci in various living human cell lines. These findings are supportive of the signal denoising effect of CRISPR/Pepper‐tDeg system mediated by the rapid degradation of both unbound sgRNA and unbound fluorogenic proteins.

Because deficiencies in telomere length have been considered as risk factors in several diseases, including cardiovascular disease (CVD), diabetes, obesity, liver cirrhosis, and cancer, the telomere length measurement is applied for the clinical diagnosis of patients.^[^ ^41^ ^]^ We therefore sought to examine the ability of CRISPR/Pepper‐tDeg for sensitive telomere length measurement. To this end, we compared the telomere images of Hela, U2OS and HEK293T cells, which demonstrated different telomere length distribution profiles in these three different cell lines (Figure S8, Supporting Information). U2OS cells had a 1.2‐fold longer telomere length (represented by the mean intensity of telomere foci) with a more dispersive distribution profile than Hela cells, which was consistent with the previously reported U2OS cells displaying longer and more heterogeneous telomere lengths than Hela cells.^[^ ^41^ ^]^ Moreover, the two cancer cell lines Hela and U2OS showed telomere lengths shorter than those in normal cell line HEK293T, as a reduced telomere length was frequently observed in cancer cell lines.^[^ ^41^ ^]^ These results suggest that CRISPR/Pepper‐tDeg not only enables sensitive telomere length measurement in single cells, but also provides a tool to estimate the heterogeneity of telomere length in cell populations, which is promising in clinical, epidemiological and research studies.

In order to broaden the application of CRISPR/Pepper‐tDeg, we examined its capability to label different repetitive sequence targets, such as centromeres, by adopting centromere‐targeted sgRNA (sgCentro‐Pepper)/mNeonGreen‐tDeg, which was further combined with sgTelo‐MS2/MCP‐BFP to realize simultaneous labeling of the centromeres and telomeres (Figure 3f). Different from the small punctate staining pattern of telomeres (Figure 3b), the centromere foci had a patchy staining pattern and were much larger in size (Figure 3g), which was consistent with the previous report using CRISPR/MB imaging approach.^[^ ^17^ ^]^ The combination with CRISPR/MS2‐MCP system, despite the higher background and nucleolar signals, allowed CRISPR/Pepper‐tDeg to label repetitive centromeres and telomeres simultaneously in HEK293T cells, which showed distinct pattern of subnuclear localization (Figure 3h). All these results indicate that CRISPR/Pepper‐tDeg enables dual‐color labeling of different genomic loci, possessing high compatibility with the conventional methods. Moreover, as previous studies had demonstrated that the color palette of the tDeg‐tagged fluorescent proteins can be expanded,^[^ ^33^, ^34^, ^42^ ^]^ CRISPR/Pepper‐tDeg system has great potential in multicolor imaging of genomic loci with different fluorogenic proteins.

### 2.3 Real‐Time Tracking of Repetitive Genomic Loci in Living Cells

**Figure 4:**
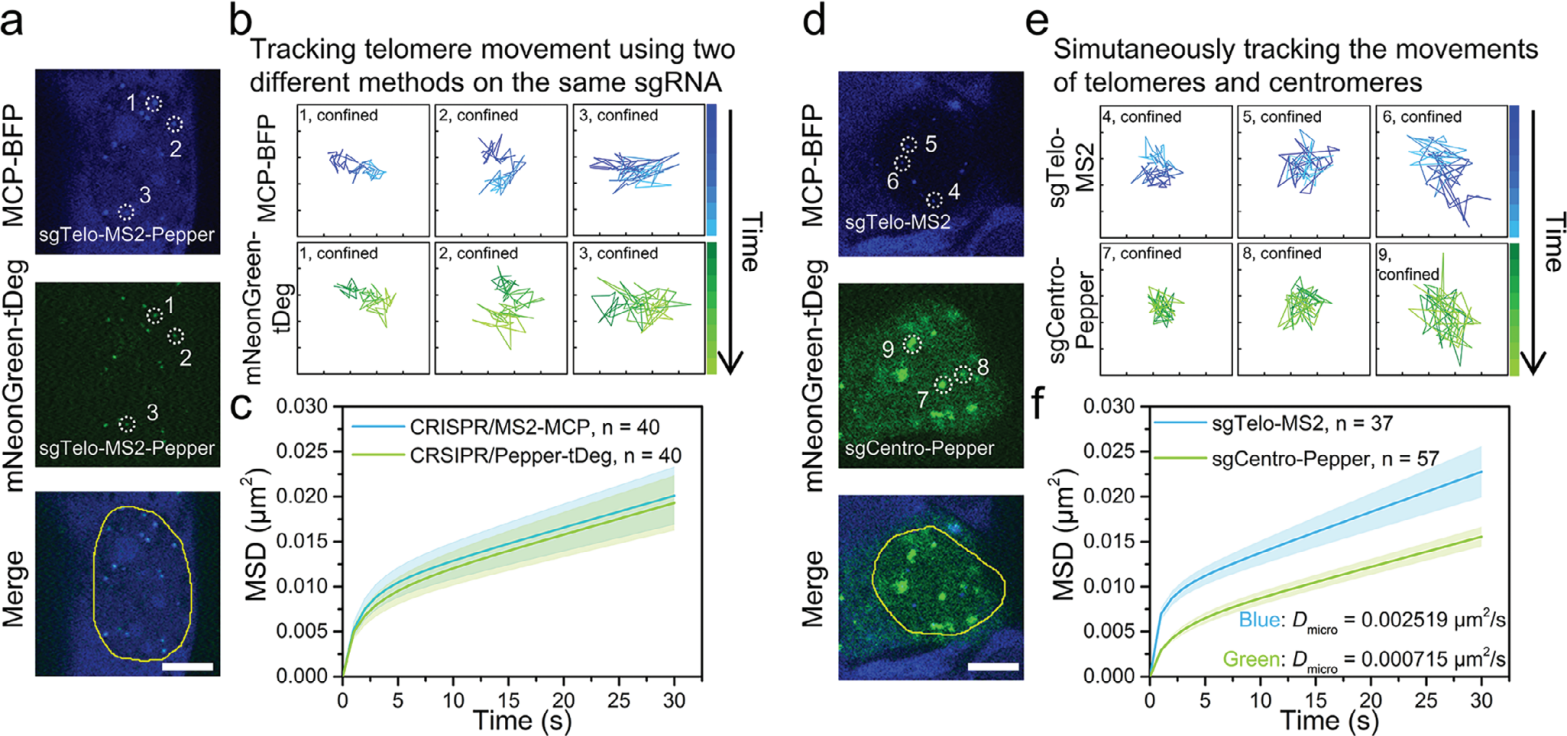
Dual‐color real‐time tracking of repetitive genomic loci in living HEK293T cells. a) Representative confocal images of telomeres co‐labeled with the MCP‐BFP (blue) and mNeonGreen‐tDeg (green) using one sgTelo‐MS2‐Pepper. Tracking of individual telomeres was marked by dash circles. Scale bar: 5 µm. b) The tracking trajectories of three marked telomeres in (a), blue trajectories for the MCP‐BFP channel and green trajectories for the mNeonGreen‐tDeg channel. c) Averaged MSD curves of telomere trajectories using CRISPR/MS2‐MCP (blue) and CRISPR/Pepper‐tDeg (green). d) Dual‐color simultaneous labeling of telomeres (MCP‐BFP, blue) and centromeres (mNeonGreen‐tDeg, green) using sgTelo‐MS2 and sgCentro‐Pepper, respectively. Tracking of individual telomeres and centromeres was marked by dash circles. Scale bar: 5 µm. e) The tracking trajectories of three telomeres (blue) and three centromeres (green) marked in (d). f) Averaged MSD curves of telomeres labeled by CRISPR/MS2‐MCP (blue) and centromeres labeled by CRISPR/Pepper‐tDeg (green). Every trajectory square was 500 nm × 500 nm on (b) and (e). The trajectory lengths were 60 frames with a time interval of 3.313 s. Data in (c) and (f) are means ± SE.

We first used the sgRNA carrying MS2‐Pepper loops (i.e., sgTelo‐MS2‐Pepper) to track and compare the dynamics of individual telomeres co‐labeled with MCP‐BFP and mNeonGreen‐tDeg in living HEK293T cells (**Figure** **4**a). Regardless of the lag of capturing images between two channels (blue and green), trajectories of two channels within a duration about 199 seconds were similar revealed by single‐particle tracking analysis, both showing the confined diffusion of telomere motion (Figure 4b, Supplementary Movies S1 and S2, Supporting Information). The corresponding mean‐squared displacement (MSD) analysis of telomeres gave similar curves and diffusion coefficients for both channels (Figure 4c). Next, we tested the dual‐color tracking of telomeres and centromeres by sgTelo‐MS2/MCP‐BFP and sgCentro‐Pepper/mNeonGreen‐tDeg in living HEK293T cells. The telomeric and centromeric foci displayed unique motion with different size, probably ascribed to their different lengths (Figure 4d,e, Movies S3 and S4, Supporting Information).^[^ ^6^ ^]^ The recorded movements of telomeres and centromeres were fitted with walking confined diffusion, which showed different microscopic diffusion coefficients *D*_micro_ (Figure 4f).^[^ ^12^ ^]^ Interestingly, we observed a relatively lower *D*_micro_ for centromeres (green), representing a slower microscopic diffusion speed than telomeres (blue), possibly because centromeres were trapped in interphase cells.^[^ ^12^ ^]^ CRISPR/Pepper‐tDeg can therefore realize dual‐color and simultaneous imaging of different genomic loci in living cells.

### 2.4 Signal Amplification by Split Fluorescent Proteins for Labeling Non‐Repetitive Sequences

**Figure 5:**
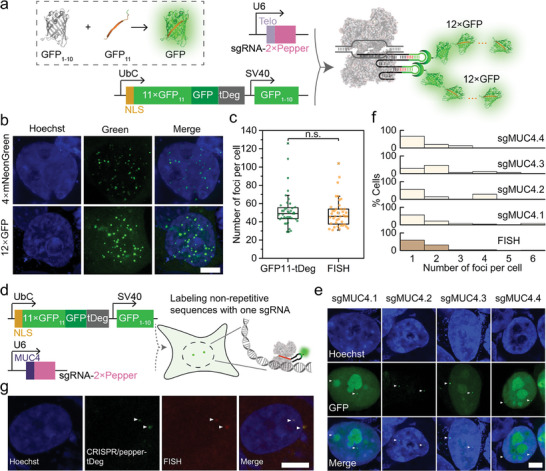
Signal amplification of CRISPR/Pepper‐tDeg by split GFP design. a) Schematic diagram of split‐GFP‐coupled CRISPR/Pepper‐tDeg system. b) Labeling of telomeres by CRISPR/Pepper‐tDeg using NLS‐(mNeonGreen)_4_‐tDeg and NLS‐GFP11‐tDeg. Images are maximum z projections. Scale bar: 5 µm. c) Box plot showing the total number of telomere foci detected per cell using NLS‐GFP11‐tDeg and FISH; n  =  40 cells for both. d) Schematic diagram of split‐GFP‐coupled CRISPR/Pepper‐tDeg system for labeling non‐repetitive genomic loci. e) Labeling of non‐repetitive MUC4 by CRISPR/Pepper‐tDeg using sgMUC4.1‐, sgMUC4.2‐, sgMUC4.3‐ and sgMUC4.4‐Pepper. Images are maximum z projections. Scale bar: 5 µm. f) Histograms of MUC4 loci counts by split‐GFP‐coupled CRISPR/Pepper‐tDeg labeling and FISH. g) Co‐labeling of MUC4 by split‐GFP‐coupled CRISPR/Pepper‐tDeg (green) and FISH (red). Scale bar: 5 µm. n.s., not significant using two‐tailed *t*‐test.

To achieve sufficient SNR when using one sgRNA for the labeling of non‐repetitive genomic loci, in which only one copy of dCas9‐sgRNA complex can be anchored,^[^ ^31^ ^]^ we attempted to develop a signal amplification on the CRISPR/Pepper‐tDeg system. Generally, signal amplification, which can be achieved by either inserting dozens of RNA aptamers into sgRNA or adopting a tandem of fluorescent proteins, is still challenging because plenty of RNA aptamer insertions reduced the sgRNA expression seriously^[^ ^16^ ^]^ and more than three copies of fluorescent protein fusion caused difficulties to plasmid construction from bacterial recombination,^[^ ^24^, ^42^ ^]^ respectively. Therefore, we selected the split GFP system as a signal amplifier of the CRISPR/Pepper‐tDeg by using a GFP11‐tDeg plasmid to express a GFP‐tDeg fusion tagged with a tandem of GFP11 (the eleventh *β*‐strand with only 16 amino acids) and a GFP1‐10 fragment (the first to the tenth *β*‐strands), which can be reconstituted with each other and form the fluorescent GFP.^[^ ^42^, ^43^, ^44^ ^]^ The split GFP system using GFP11‐tDeg under the control of miniCMV was first tested in the Pepper‐tDeg system and compared with (mNeonGreen)_4_‐tDeg (Figure S9, Supporting Information). Both the FP‐tDeg fusion led to a negligible background fluorescence in cells without Pepper expressing (Figure S9a, Supporting Information). As expected, (mNeonGreen)_4_‐tDeg and GFP11‐tDeg with varying degrees of signal amplification (4× and 12×, respectively) induced increasing fluorescence in circular‐Pepper‐expressing HEK293T cells since the repeats of fluorescent protein tandem increased (Figure S9a, Supporting Information). Further confirmed by flow cytometry analysis, the SNR increased with increasing degree of signal amplification, which was 4.7 and 16.0 for 4× and 12× signal amplification, respectively (Figure S9b, Supporting Information). Furthermore, the GFP11‐tDeg with the UbC promoter and NLS insertion was used to label telomeres with high efficiency (**Figure** **5**a–c), which increased the observable number of telomeres per cell by 1.85‐ and 1.25‐fold, as well as the labeled cell numbers by 2.50‐ and 1.74‐fold as compared to the (mNeonGreen)_4_‐tDeg without or with the UbC promoter and NLS insertion, respectively (Figure 2c). These results reveal that the split‐GFP‐coupled CRISPR/Pepper‐tDeg system successfully achieved a further improved imaging efficiency, which was promising for labeling a non‐repetitive genomic locus using only one sgRNA. In order to verify that this labeling efficiency was sufficiently high, the total number of telomeres identified by the split‐GFP‐coupled CRISPR/Pepper‐tDeg system or standard FISH (Figure S10, Supporting Information) was measured and compared. As shown in Figure 5c, the numbers identified by these two methods are well matched, revealing a similar labeling efficiency of split‐GFP‐coupled CRISPR/Pepper‐tDeg with FISH.

Next, the capacity of split‐GFP‐coupled CRISPR/Pepper‐tDeg system to label non‐repetitive genomic loci was examined by transfecting HEK293T cells with a sgRNA‐Pepper targeting non‐repetitive MUC4.1, MUC4.2, MUC4.3, or MUC4.4 (Figure 5d). All sgRNAs were capable to target the non‐repetitive region of MUC4 (Figure 5e,f). Frequently we observed 2 – 3 labeled MUC4 loci per HEK293T cell, which was consistent with the previous report.^[^ ^6^, ^27^, ^31^ ^]^ The occasional appearance of 4 – 6 copies of MUC4 per cell might be attributed to nonspecific labeling from the off‐target affinity exhibited in the CRIPSR‐dCas9 system, which generally happens if fewer sgRNAs are used (Figure 5f).^[^ ^29^, ^36^ ^]^ To assess the labeling specificity of split‐GFP‐coupled CRISPR/Pepper‐tDeg system, we co‐labeled the MUC4 with a Cy5‐MUC4 DNA FISH probe^[^ ^29^ ^]^ targeting the repetitive regions of MUC4 in HEK293T cells transfected with MUC4.1‐sgRNA‐Pepper (Figure 5g). The well co‐localization with DNA FISH proved that the split‐GFP‐coupled CRISPR/Pepper‐tDeg enables highly specific labeling of non‐repetitive loci with one sgRNA. We continued to examine whether the split‐GFP‐coupled CRISPR/Pepper‐tDeg system can be applied to label other genomic loci including non‐repetitive sequence in living cells with one sgRNA. To this end, HEK293T cells were transfected with the system along with a sgRNA targeting either repetitive C3 (∼500 copies),^[^ ^33^ ^]^ IDR1 (61 copies),^[^ ^16^ ^]^ IDR3 (45 copies),^[^ ^16^ ^]^ FBN3 (22 copies)^[^ ^16^ ^]^ or non‐repetitive IL‐1B.^[^ ^31^ ^]^ Consistent with the previous reports, two to three foci were detected for these sequences (Figure S11, Supporting Information).^[^ ^16^, ^31^, ^33^ ^]^ These results confirm that the proposed system is able to specifically label different repetitive and non‐repetitive genomic loci with diverse copies.

Our CRISPR/Pepper‐tDeg system is consisted of one unique engineered sgRNA to carry two fluorogenic tandem split GFP, and theoretically can recruit as many as 2 × 12 copies of GFP at a target non‐repetitive genomic locus anchored with one copy of dCas9‐sgRNA complex, achieving 24× signal amplification while without background noise distraction as the unbound sgRNA and unbound fluorogenic tandem split GFP can be fast degraded. The appropriate nuclear expression and the fast degradation of fluorogenic split GFP are the key to improve SNR in this CRISPR/Pepper‐tDeg system, allowing the efficient labeling of non‐repetitive genomic loci using one sgRNA engineered with only 2 × Pepper aptamers. This readily accessible feature is an advantage over most CRISPR‐based imaging techniques that heavily rely on tedious modifications of dCas9 (e.g., with 24 × Suntag)^[^ ^23^ ^]^ or sgRNA (e.g., with 14 × MS2)^[^ ^14^ ^]^ to carry constitutively fluorescent proteins as many as possible for improving SNR, resulting in decreased expression/stability and thus a reduced labeling efficiency, and especially suffering from the high “always‐on” background signal (Table S3, Supporting Information). In comparison, the minimalist design of CRISPR/Pepper‐tDeg system using fluorogenic split GFP protein for signal amplification avoids or minimizes the loss in expression/stability of each component, thus holding a robust labeling efficiency while drastically denoising background signal.

Moreover, sensitive imaging of non‐repetitive genomic loci using only one sgRNA, which remains a huge challenge, is still the focus of intense research in this field. Until now, there are only three latest techniques including CRISPR/Casilio,^[^ ^29^ ^]^ CRISPR FISHer^[^ ^30^ ^]^ and CRISPR SIMBA^[^ ^31^ ^]^ making this effort, which still have such technical difficulties as extensive sgRNA modifications reducing the labeling efficiency, and complex plasmid constructions precluding the broad applications (Table S3, Supporting Information). In comparison, our CRISPR/Pepper‐tDeg system with one sgRNA has such distinct features as the minimalist design, easy operation, and comparable accuracy with gold standard FISH. Whereas, due to the off‐target affinity exhibited in the CRIPSR‐dCas9 system,^[^ ^29^ ^]^ the nonspecific labeling issue needs to be carefully assessed and addressed in this CRISPR/Pepper‐tDeg system when only one sgRNA is used, which is a common issue for all these methods using fewer sgRNAs.^[^ ^36^ ^]^ Future studies will be required to increase the targeting specificity of sgRNA through engineering dCas9 variants with reduced off‐target affinity.

Overall, benefiting from these abovementioned unique merits, the CRISPR/Pepper‐tDeg system is easy to be mastered and popularized for quantifying repetitive and even non‐repetitive genomic loci in living cells, showing promising utility in routine clinical diagnosis such as the diagnosis of telomere length deficiency‐related diseases, and thus demonstrating the large application potential of this genomic‐imaging system in biological research, clinical diagnosis and therapeutic evaluation.

## 3 Conclusion

In summary, we have developed a CRISPR/Pepper‐tDeg genomic imaging system for quantitative analysis of repetitive and even non‐repetitive sequences, as well as for real‐time, dual‐color tracking of genomic loci in living cells. In contrast to the current genomic‐imaging systems using “always‐on” fluorescent protein tag on either dCas9 or sgRNA, the CRISPR/Pepper‐tDeg, by taking advantage of the target‐dependent stability switch of fluorogenic CRISPR−protein complexes, possesses two crucial merits: (1) High SNR with a 5‐fold enhancement based on the dual signal denoising and simultaneously, dual signal amplification. The signal denoising by the rapid degradation of unbound sgRNA and fluorogenic proteins, along with the signal amplification by one sgRNA carrying modest Pepper aptamer loops tagged with fluorescent protein tandem, allow the CRISPR/Pepper‐tDeg to label and track repetitive telomeres and centromeres with high efficiency and specificity; (2) Facile design for achieving non‐repetitive sequence imaging, which is easy to popularize. Without the need of abundant sgRNAs transfection or numerous RNA aptamers insertion, the split‐GFP‐coupled CRISPR/Pepper‐tDeg system obtained a further improved SNR, which successfully achieved robust and specific labeling of a non‐repetitive genomic locus using only one sgRNA with two aptamer insertions.

Notably, a similar fluorogenic CRISPR (fCRISPR) method has been reported very recently to address high background and detect repetitive genomic loci with more than 14 copies.^[^ ^33^ ^]^ In comparison, our CRISPR/Pepper‐tDeg system aims at simple yet robust labeling of the non‐repetitive genomic loci using one sgRNA, which critically relies on two key factors—the appropriate expression of FP‐tDeg in nucleus and the efficient signal amplification manner without noise magnification. On the shared basis of fluorogenic protein technology developed recently,^[^ ^34^ ^]^ we therefore made the first attempt to optimize the promoter and add NLS to the FP‐tDeg fusion, resulting in an appropriate expression level and nuclear localization of FP‐tDeg. Notably, to compare CRISPR/Pepper‐tDeg with conventional CRISPR/MS2‐MCP homogeneously, we also made the first attempt to fuse two aptamers, MS2 and Pepper, to stem loops of the same sgRNA. This attempt allows the direct SNR calculation and comparison on the same telomeres co‐labeled by CRISPR/MS2‐MCP and CRISPR/Pepper‐tDeg in the same cells without cell heterogeneity, unlike the conventional comparison achieved in different cells transfected with different systems. With optimization of the FP‐tDeg fusion, the SNR had a 5‐fold (via homogeneous calculation) enhancement compared with conventional CRISPR/MS2‐MCP, which can be further improved significantly after coupled with the split fluorescent protein design for signal amplification. After the two key factors were fulfilled, our CRISPR/Pepper‐tDeg system, as one of the few successful examples, enables the direct visualization of the nonrepetitive genomic loci, including the non‐repetitive MUC4 and IL‐1B, using only one sgRNA. The high labeling consistency with standard FISH indicates that CRISPR/Pepper‐tDeg is a robust and efficient method comparable to standard FISH for detecting non‐repetitive genomic loci, with the extra feature of live‐cell labeling. Besides the abovementioned metrics, the CRISPR/Pepper‐tDeg also shows high complementarity with the current genomic‐labeling approaches, thus combining these methods provides great opportunity for simultaneous imaging of multiplex genomic loci in living cells. With the evolution of the fluorogenic protein technology, we believe that this fluorogenic design will enormously promote the development of CRISPR‐based imaging systems for diverse genomic loci, which is highly helpful to the thorough understanding of chromosome regulatory mechanism, the pathogenesis and pathological process of genetic diseases.

## 4 Experimental Section

### Materials and Plasmids Construction

All PCRs were performed with 2 × TransStart FastPfu Fly PCR SuperMix (TransGen #AS231). All cloning competent cells including DH5α, Stbl3 and DB3.1 were purchased from TransGen. All the DNA sequences were synthetized by Genewiz (Suzhou, China). miniCMV‐(mNeonGreen)_4_‐tDeg (Addgene #129402) and pAV‐U6+27‐Tornado‐F30‐Pepper (Addgene #129405) were gifts from Samie Jaffrey, GFP_1‐10_‐miniCMV‐GFP_11_×11‐GFP‐tDeg (Addgene #185404) was a gift from Jianhui Jiang. To optimize the promoters, UbC and EFS promoters were cloned into the MluI and HindIII sites to replace miniCMV promoter in miniCMV‐(mNeonGreen)_4_‐tDeg, named UbC‐(mNeonGreen)_4_‐tDeg and EFS‐(mNeonGreen)_4_‐tDeg, respectively. To optimize nuclear localization, mNeonGreen was replaced by NLS‐mNeonGreen between HindIII and KpnI sites in UbC‐(mNeonGreen)_4_‐tDeg, named UbC‐NLS‐(mNeonGreen)_4_‐tDeg. To make a comparison with CRISPR/MS2‐MCP, the MCP‐3 × BFP‐NLS segment from pHAGE‐EFS‐MCP‐3 × BFP‐NLS (Miaoling #P13647) was cloned into the XbaI and HindIII sites in UbC‐(mNeonGreen)_4_‐tDeg, named UbC‐MCP‐(BFP)_3_‐NLS. To label non‐repetitive sequences, GFP_1‐10_ and NLS‐GFP_11_ × 11‐GFP‐tDeg amplified from GFP_1‐10_‐miniCMV‐GFP_11_ × 11‐GFP‐tDeg were inserted into the HindIII and XbaI sites, SmaI and BstBI sites in UbC‐(mNeonGreen)_4_‐tDeg, respectively. This combined plasmid was named UbC‐NLS‐11 × GFP_11_‐GFP‐tDeg‐SV40‐GFP_1‐10_‐NLS. To further improve the transfection efficiency, U6‐sgRNA was combined into the BglII and MluI sites of UbC‐FP‐tDeg. The pHAGE‐TO‐dCas9 plasmids (Miaoling #P0906) was a gift from Thoru Pederson. T2A‐Blastin was cloned into BamHI and XbaI sites in pHAGE‐TO‐dCas9, and used for subsequent construction of HEK293T/dCas9 cells, Hela/dCas9 cells and U2OS/dCas9 cells (obtained from Editgene, Guangzhou, China) by blasticidin selection. CcdB was amplified from pLH‐sgRNA1‐2 × MS2 (Miaoling #P14585) and then inserted into the BbsI sites of PspCas13b crRNA backbone (Miaoling #P2166). All the modified sgRNAs were then cloned into the SbfI and KpnI sites to compose ccdB‐sgRNA. All the target spacers were inserted to the desired ccdB‐sgRNA. All modified sgRNA sequences were listed in Table S1. All plasmids used in different experiments were listed in Table S2.

### Cell Culture and Transfection

HEK293T cells (Procell #CL‐0005) and HEK293T/dCas9 cells were cultured in Dulbecco's Modified Eagle's Medium (DMEM) (Gibco, ThermoFisher Scientific #C11995500BT) with 10% fetal bovine serum (FBS) (PAN #ST30‐3302), and 1% penicillin‐streptomycin (Gibco, ThermoFisher Scientific #15140122). Hela/dCas9 cells were cultured in Minimum Essential Medium (MEM) (Procell # PM150410) with 10% FBS, and 1% penicillin‐streptomycin. U2OS/dCas9 cells were cultured in McCoy's 5A medium (Procell # PM150710) with 10% FBS, and 1% penicillin‐streptomycin. All cells were cultured at 37 °C and 5% CO_2_ in a humidified incubator. Before transfection, cells were seeded on 14 mm sterilized glass coverslips with a density of 1 × 10^5^ cells/compartment for 24 h. Then, cells were transfected with 500 ng of each plasmid using 1.5 µL Lipofectamine 3000 (Invitrogen, ThermoFisher Scientific #L3000‐008) in Opti‐MEM medium (ThermoFisher Scientific #31985070). Media were changed after 5 h. Finally, the transfected cells were incubated for another 24 h.

### qRT‐PCR

After HEK293T cells were transiently transfected with mNeonGreen‐tDeg without or with circular Pepper for 24 h, total RNA from the cells was extracted using the Total RNA Kit I (Omega #R6834) according to the manufacturer's protocol. Then, 10 ng of total RNA extract was added with 10 µL of One‐Step qRT‐PCR mixture (Vazyme #Q221‐01) containing 0.4 µM Forward Primer, 0.4 µM Reverse Primer, 1× One Step SYBR Green Mix, and 0.5 µL One Step SYBR Green Enzyme Mix. The qRT‐PCR reaction was proceeded with cDNA synthesis at 50 °C for 15 min and qPCR process conducted in LightCycler480II (Roche, USA).

### DNA FISH

FISH probes (Cy5‐MUC4: /5’Cy5/CTTCCTGTCACCGAC, TAMRA‐MUC4: /5’TAMRA/ CTTCCTGTCACCGAC) were synthesized by Sangon Biotechnology Co., Ltd. (Shanghai, China). Desired cells were first fixed with 4% formaldehyde for 10−30 min, briefly washed once with PBS, and permeabilized with 0.5% PBST for 20 min. After washed with PBS, cells were dehydrated by a series of 75%, 85% and 100% ethanol for 3 min each step. After air‐drying, 2 ng/µL MUC4 probe in hybridizing solution (10% dextran sulfate, 50% formamide, 500 ng/ml Salmon sperm DNA in 2× SSC buffer)^[^ ^45^ ^]^ was added, and incubated overnight at 37 °C in a dark humidified chamber. After hybridization, this coverslip was washed with wash buffer (2× SSC, 10% v/v formamide), then washed with 2× SSC for three times. Subsequently, cells were counterstained with 5 µg/mL Hoechst (Invitrogen, ThermoFisher Scientific #H1399) for 5 min and incubated in PBS prior to imaging.

### Flow Cytometry

HEK293T cells were seeded on 24‐well plates for 24 h, followed by transfection for another 24 h. The cells were washed with PBS, then digested by trypsin (Gibco, ThermoFisher Scientific #C25200056), washed and suspended with cooled PBS for subsequent flow cytometry measurements using CytoFLEX S (Beckman Coulter, USA). mNeonGreen was detected by FITC channel (525/40).

### Confocal Imaging

Besides cells transfected with BFP, all cells were stained with 5 µg/mL Hoechst prior to imaging. Fluorescence images were acquired on FV3000 confocal microscope (Olympus, Japan). BFP and Hoechst were excited at 405 nm with emission wavelength of 425−475 nm, mNeonGreen was excited at 488 nm with emission wavelength of 495−545 nm, TAMRA was excited at 561 nm with emission wavelength of 570−620 nm and Cy5 was excited at 640 nm with emission wavelength of 650−690 nm. BFP and mNeonGreen were acquired on different phase to avoid fluorescence crosstalk.

### Single‐particle Tracking Analysis

The movements of single telomere and centromere were recorded at 3.313 s per frame with continuous 60 frames and analyzed by the trackmate plugin of ImageJ. For each trajectory, the MSD was calculated by:

(1) $$\mathrm{MSD}\left(n\Delta t\right)=\frac{1}{N-1-n}\sum_{i=1}^{N-n-1}{\left|r\left(i\Delta t+n\Delta t\right)-r\left(i\Delta t\right)\right|}^{2}$$

where *N* is the total number of frames, *n* is the time intervals, Δ*t* is 3.313 s.

All MSD curves of telomeres and centromeres were obtained from at least 15 cells, and averaged for display. The MSD curves were fitted by:

(2) $$\mathrm{MSD}\left(t\right)=A\left(1-e^{-t/\tau }\right)+4D_{macro}t+v^{2}t^{2}$$

where *A* was the confinement area, τ was a constant, *D_macro_* was the macroscopic diffusion coefficient and *v* was the velocity of active transport.^[^ ^46^ ^]^ And *D_micro_* was calculated by *D_micro_* = *A*/4τ.

### Image Processing

FlowJo_V10 was used for FACS data analysis. ImageJ was used for all confocal images.

The SNR was calculated by:

(3) $$\mathrm{SNR}=FL_{spot}/FL_{nucleus}$$

where *FL_spot_* was the maximum fluorescent intensity at a labeled locus, and *FL_nucleus_* was the mean fluorescent intensity of nucleus excluding all the labeled locus region. All the fluorescent intensity was measured by ImageJ.

The SNR percentage in Figure 3d was calculated by:

(4) $$P_{SNR\leq 5}\%=N_{SNR\leq 5}/N_{SNR\leq 40}$$

(5) $$P_{SNR>5}\%=N_{SNR>5}/N_{SNR\leq 40}$$

where *P* was the percentage and *N* was the number of telomere foci with different SNR.

### Statistical Analysis

Sample size (*n*) for each statistical analysis was at least three unless otherwise stated. All error bars represented means ± SD. All statistical analyses and *p* values are presented in the figure legends. Statistical significance was performed using two‐tailed *t*‐test to compare two groups in Excel. n.s. = not significant, *p < 0.05, **p < 0.01, ***p < 0.001 and ****p < 0.0001.

## Conflict of Interest

The authors declare no conflict of interest.

## Supporting information

Supporting Information

Supplemental Movie 1

Supplemental Movie 2

Supplemental Movie 3

Supplemental Movie 4

## Data Availability

The data that support the findings of this study are available from the corresponding author upon reasonable request.
