# Supplementary material for: CRISPR/Pepper‐tDeg: A Live Imaging System Enables Non‐Repetitive Genomic Locus Analysis with One Single‐Guide RNA
Source: Adv Sci (Weinh). 2024 Jun 26;11(32):2402534. doi: 10.1002/advs.202402534 (PMC11348139; doi:10.1002/advs.202402534)
Supplement: Supplementary file 1 — Supporting Information [file ADVS-11-2402534-s004.pdf]

ADVANCED SCIENCE
Open Access

# Supporting Information

for *Adv. Sci.*, DOI 10.1002/advs.202402534

CRISPR/Pepper-tDeg: A Live Imaging System Enables Non-Repetitive Genomic Locus Analysis with One Single-Guide RNA

*Meng Chen, Xing Huang, Yakun Shi, Wen Wang, Zhan Huang, Yanli Tong, Xiaoyong Zou, Yuzhi Xu* and Zong Dai**

Supporting Information

**CRISPR/Pepper-tDeg: A Live Imaging System Enables Non-Repetitive Genomic Locus Analysis with One Single-Guide RNA**

*Meng Chen, Xing Huang, Yakun Shi, Wen Wang, Zhan Huang, Yanli Tong, Xiaoyong Zou, Yuzhi Xu*, Zong Dai**

M. Chen, X. Huang, Y. Shi, Y. Tong, Z. Dai
Guangdong Provincial Key Laboratory of Sensing Technology and Biomedical Instrument, School of Biomedical Engineering, Shenzhen Campus of Sun Yat-Sen University, Sun Yat-Sen University, Shenzhen 518107, China
E-mail: daizong@mail.sysu.edu.cn

W. Wang
School of Pharmaceutical Sciences, Sun Yat-Sen University, Guangzhou 510275, China

Z. Huang, X. Zou
School of Chemistry, Sun Yat-Sen University, Guangzhou 510275, China

Y. Xu
Scientific Research Center, The Seventh Affiliated Hospital, Sun Yat-Sen University, Shenzhen 518107, China
E-mail: xuyzh28@mail.sysu.edu.cn

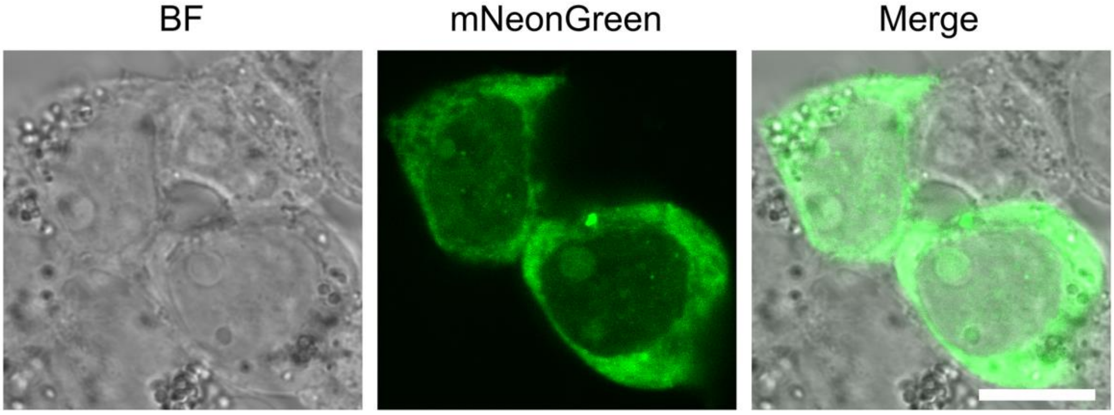


**Figure S1.** Labeling of telomeres by CRISPR/Pepper-tDeg using miniCMV-(mNeonGreen)$_4$-tDeg and Telo-sgRNA-Pepper. Scale bar: 10 μm.

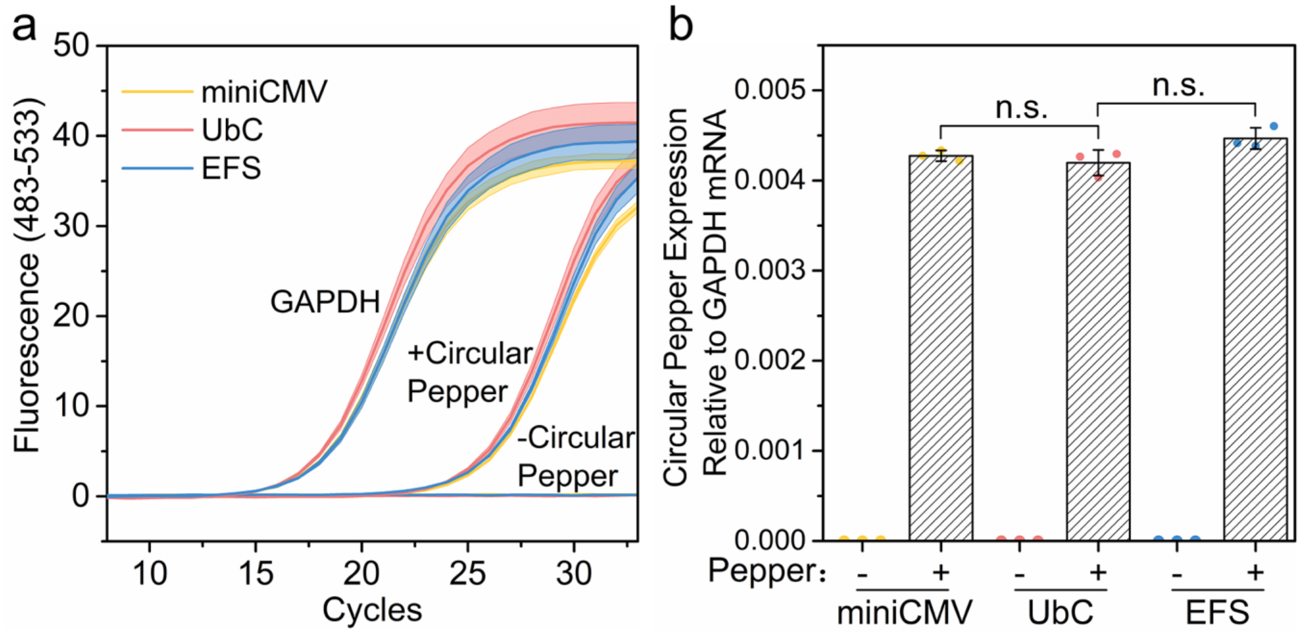


**Figure S2.** Relative expression level of circular Pepper after transfection of mNeonGreen-tDeg driven by miniCMV, UbC and EFS with or without Tornado plasmids. (a) qRT-PCR data of circular Pepper in the three mNeonGreen-tDeg-expressing groups with or without Tornado plasmid transfection. (b) Relative expression level of circular Pepper to GAPDH among the three different promoter-driven mNeonGreen-tDeg-expressing groups. Data are means ± SD, *n* = 3. n.s., not significant using two-tailed *t*-test.

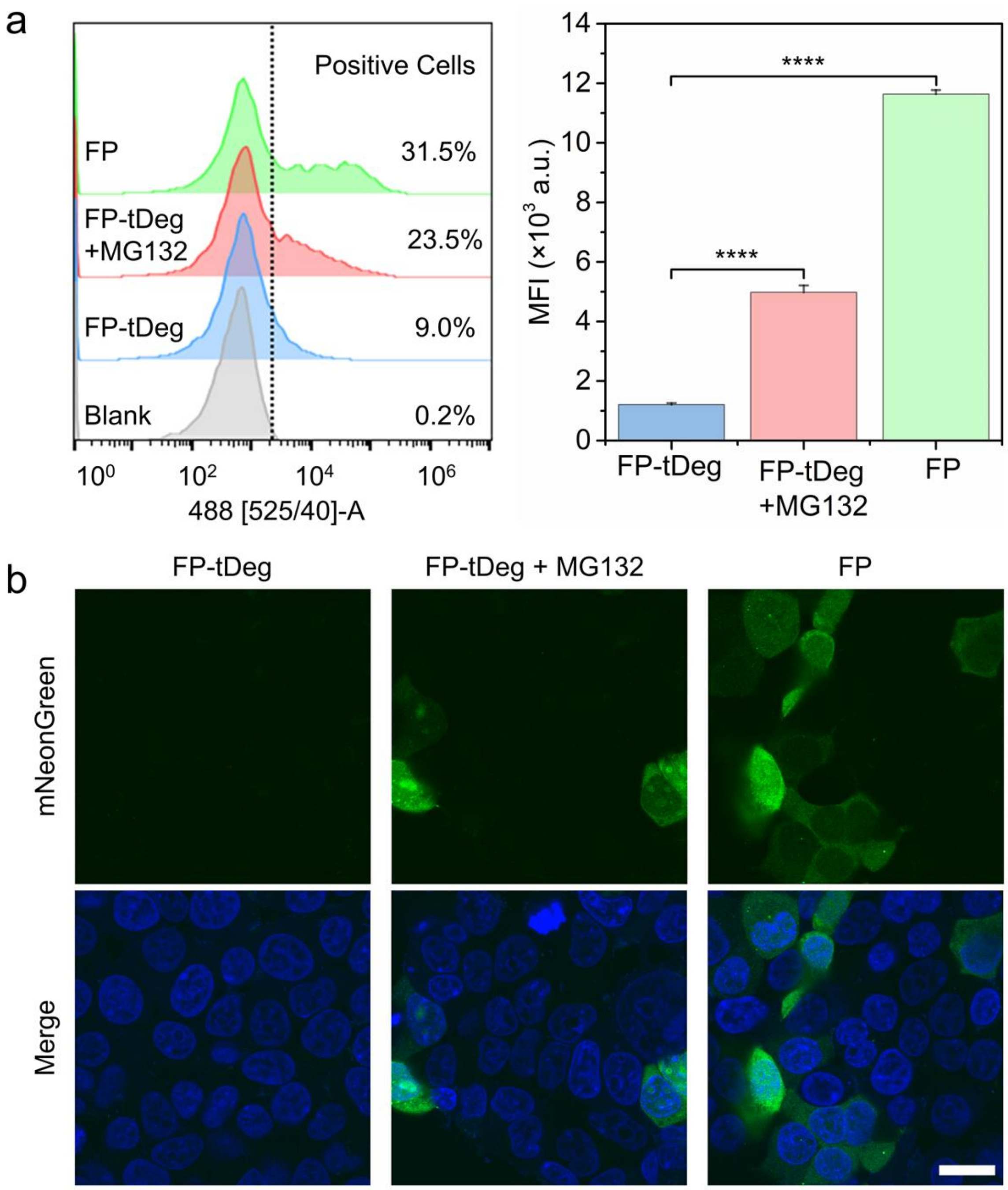


**Figure S3.** The proteasomal degradation of mNeonGreen-tDeg proved by its fluorescence recovery with the addition of proteasome inhibitor MG132. (a) Flow cytometry profiles and (b) confocal images for HEK293T cells expressing miniCMV-(mNeonGreen)$_4$, or miniCMV-(mNeonGreen)$_4$-tDeg with DMSO or 10 μM MG132. Scale bar: 20 μm. Data are means ± SD, $n = 3$. ****$p < 0.0001$ using two-tailed $t$-test.

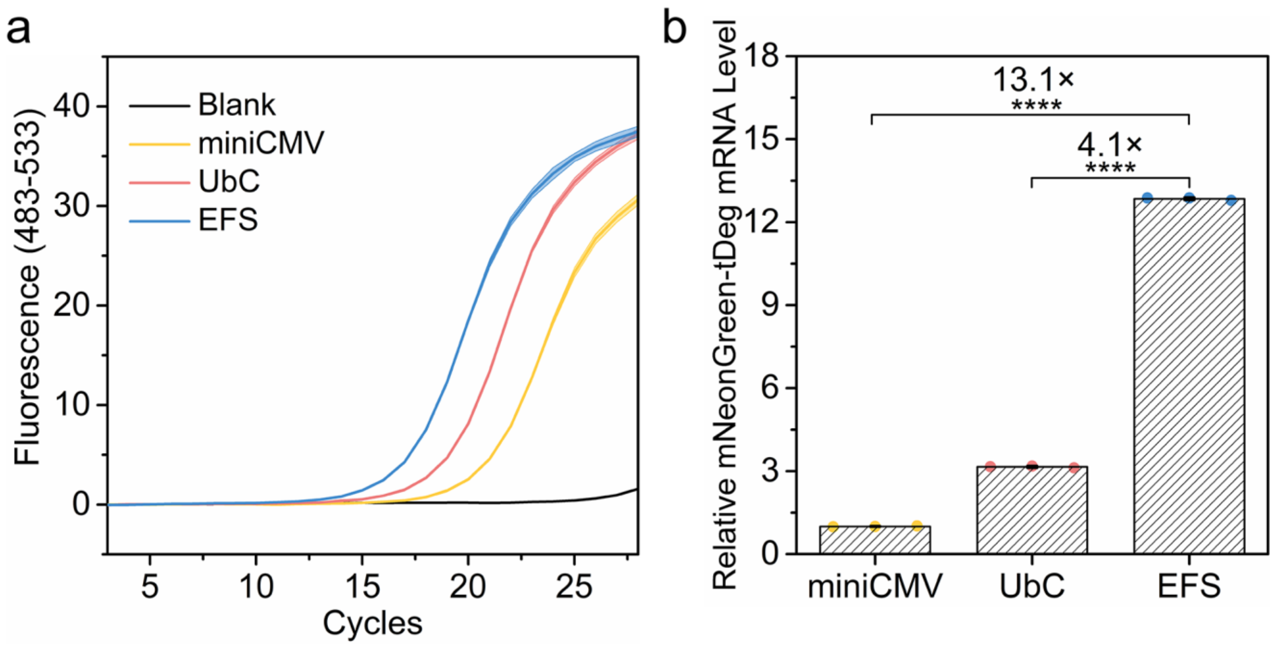


**Figure S4.** Relative expression level of mNeonGreen-tDeg mRNA with or without transfection of mNeonGreen-tDeg. (a) qRT-PCR data of mNeonGreen-tDeg under the control of miniCMV, UbC, and EFS promoters. (b) Relative expression level of mNeonGreen-tDeg mRNA. Data are means ± SD, $n = 3$. ****$p < 0.0001$ using two-tailed $t$-test.

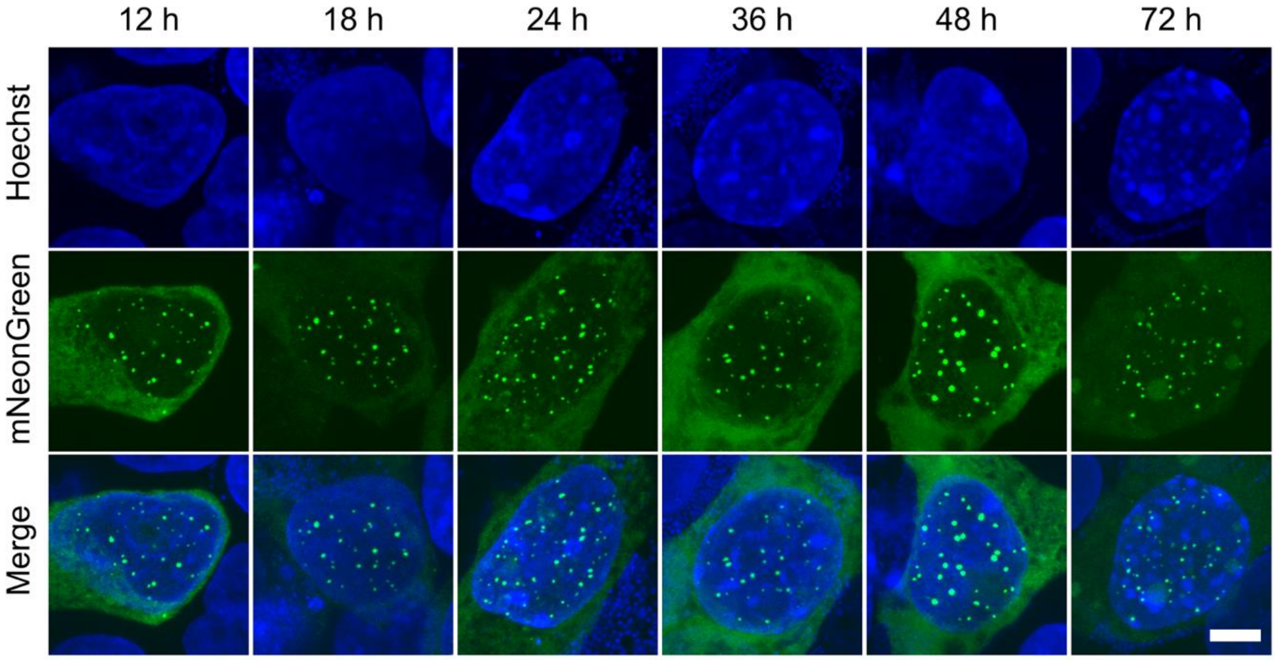


**Figure S5.** Labeling of telomeres by CRISPR/Pepper-tDeg at different time points after transfection. Images are maximum z projections. Scale bar: 5 μm.

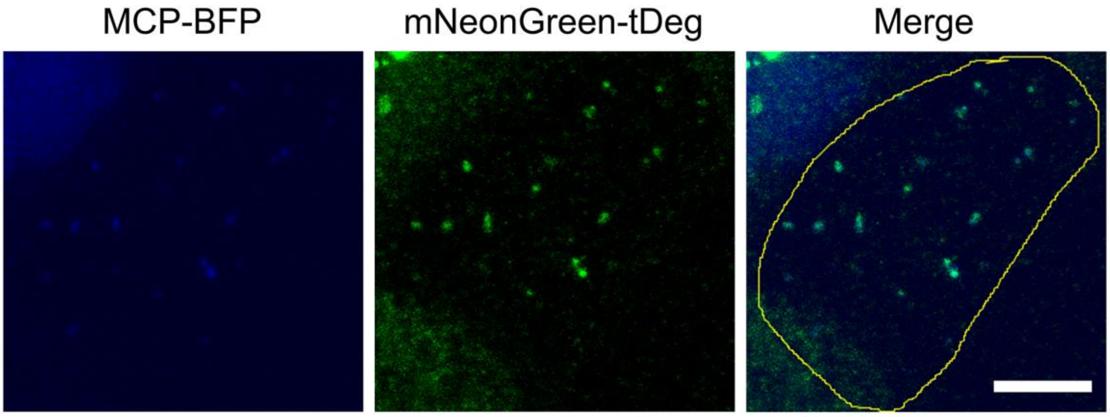


**Figure S6.** Co-labeling of telomeres by CRISPR/Pepper-tDeg and CRISPR/MS2-MCP using sgTelo-Pepper/mNeonGreen-tDeg and sgTelo-MS2/MCP-BFP, respectively. Images are maximum z projections. Scale bar: 5 μm.

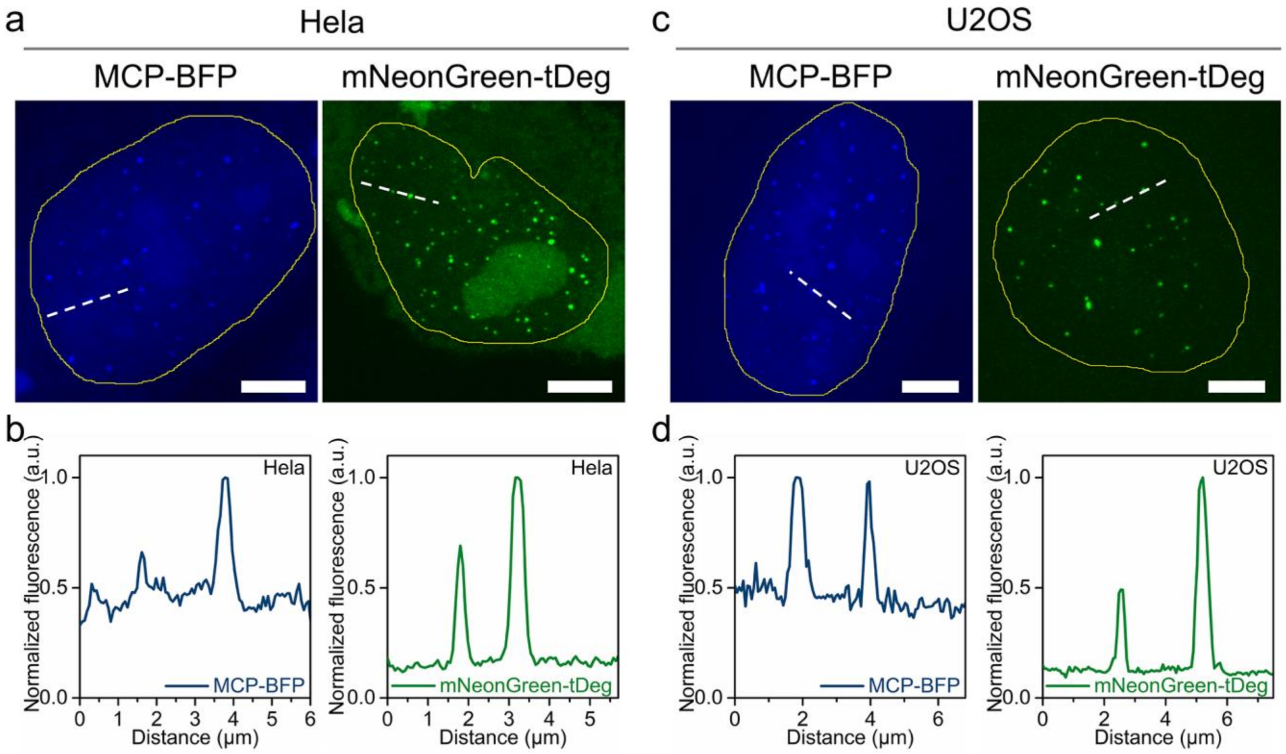


**Figure S7.** Comparison of telomere imaging between conventional CRISPR/MS2-MCP and CRISPR/Pepper-tDeg in different human cell lines. (a, b) Comparison of telomere labeling (dash line) between conventional CRISPR/MS2-MCP (left) and CRISPR/Pepper-tDeg (right) in Hela cells. (c, d) Comparison of telomere labeling (dash line) between conventional CRISPR/MS2-MCP (left) and CRISPR/Pepper-tDeg (right) in U2OS cells. Images are maximum z projections. Scale bar: 5 μm.

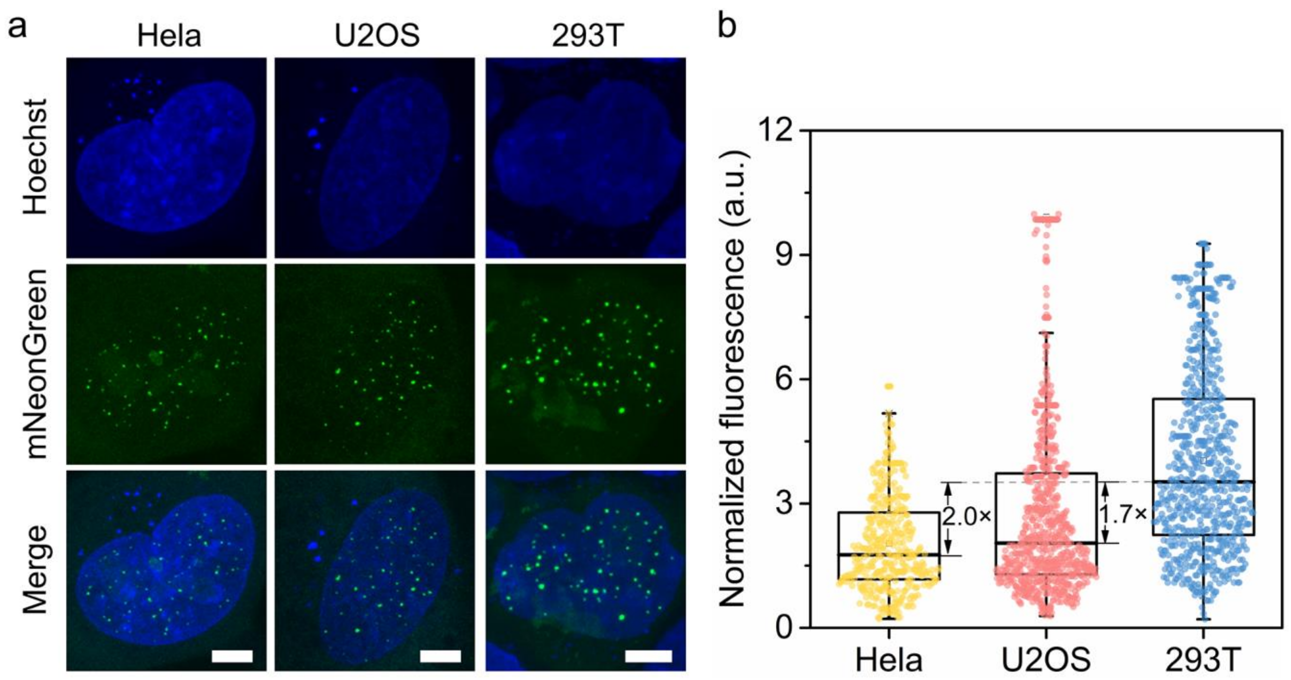


**Figure S8.** The telomere length measurement by CRISPR/Pepper-tDeg. (a) Comparison of telomere length in Hela, U2OS and 293T cells using CRISPR/Pepper-tDeg. Images are maximum z projections. Scale bar: 5 μm. (b) Distribution of individual telomere lengths represented by the normalized fluorescence of telomere foci labeled by CRISPR/Pepper-tDeg per cell; $n \geq 10$ cells.

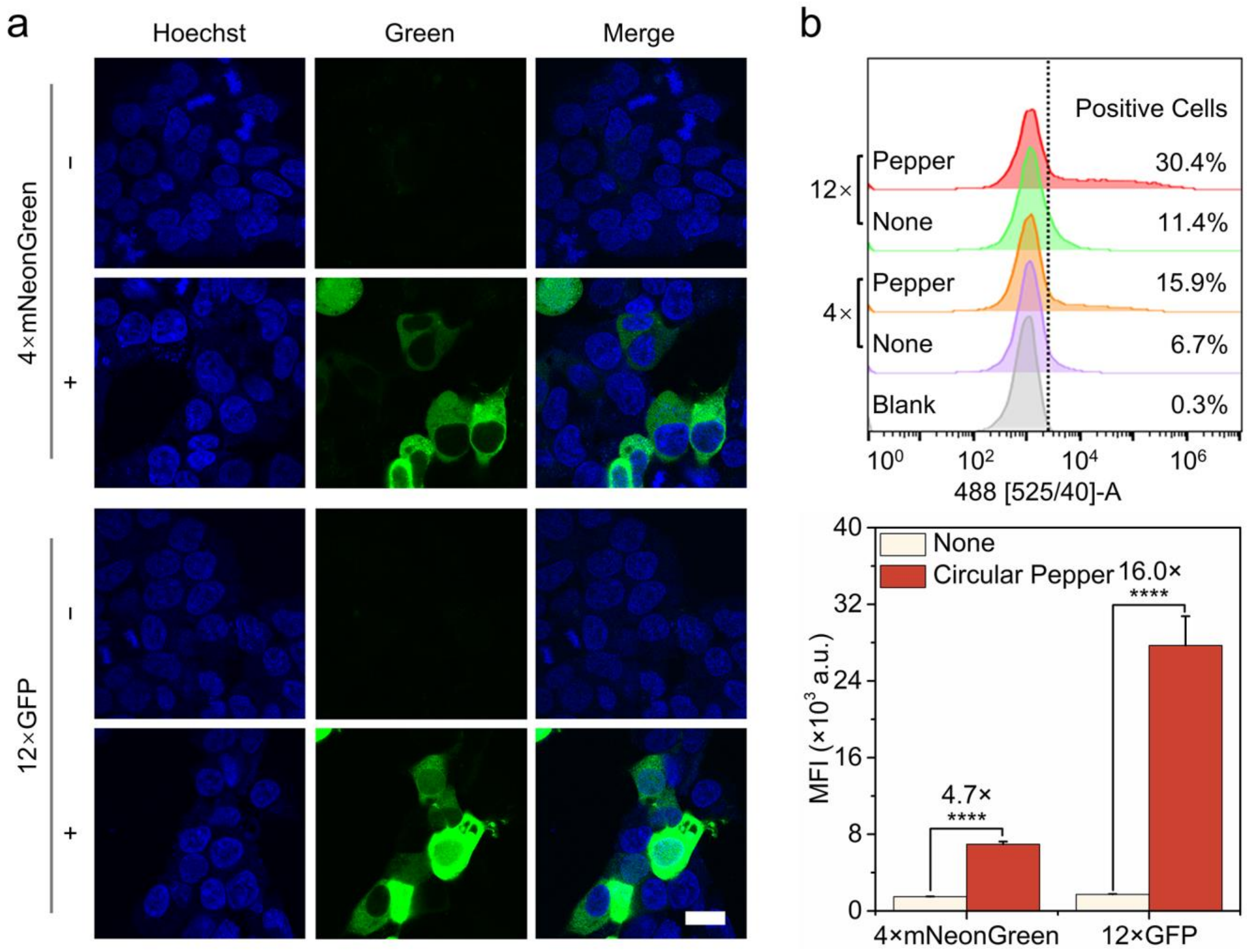


**Figure S9.** Fluorescence signal amplification by increasing repeats of fluorescent protein tag. (a) Confocal images for HEK293T cells expressing $(mNeonGreen)_4$-tDeg and GFP11-tDeg under the control of miniCMV promoters with none (−) or circular Pepper (+). Scale bar: 20 μm. (b) Flow cytometry profiles and the mean fluorescence intensity (MFI) of corresponding cells. Data are means ± SD, $n = 3$. ****$p < 0.0001$ using two-tailed $t$-test.

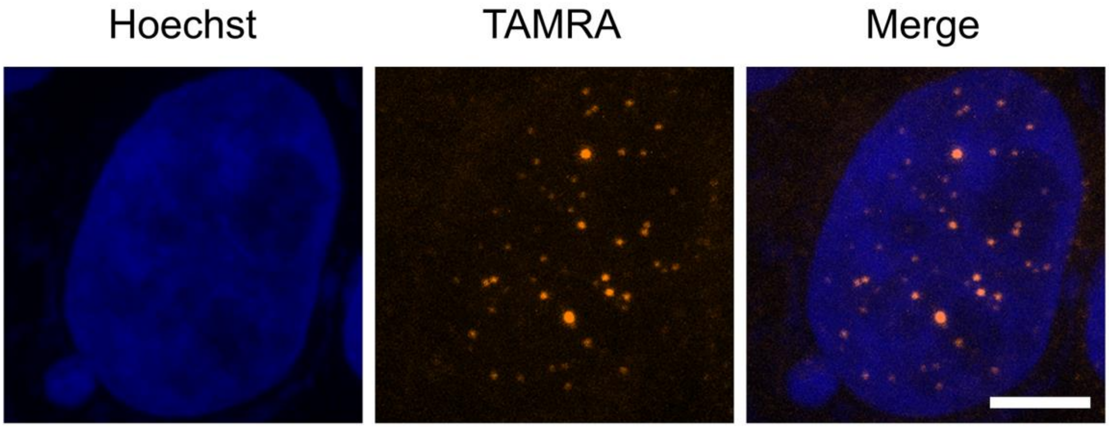


**Figure S10.** Labeling of telomeres by standard FISH in HEK293T cells. Images are maximum z projections. Scale bars: 5 μm.

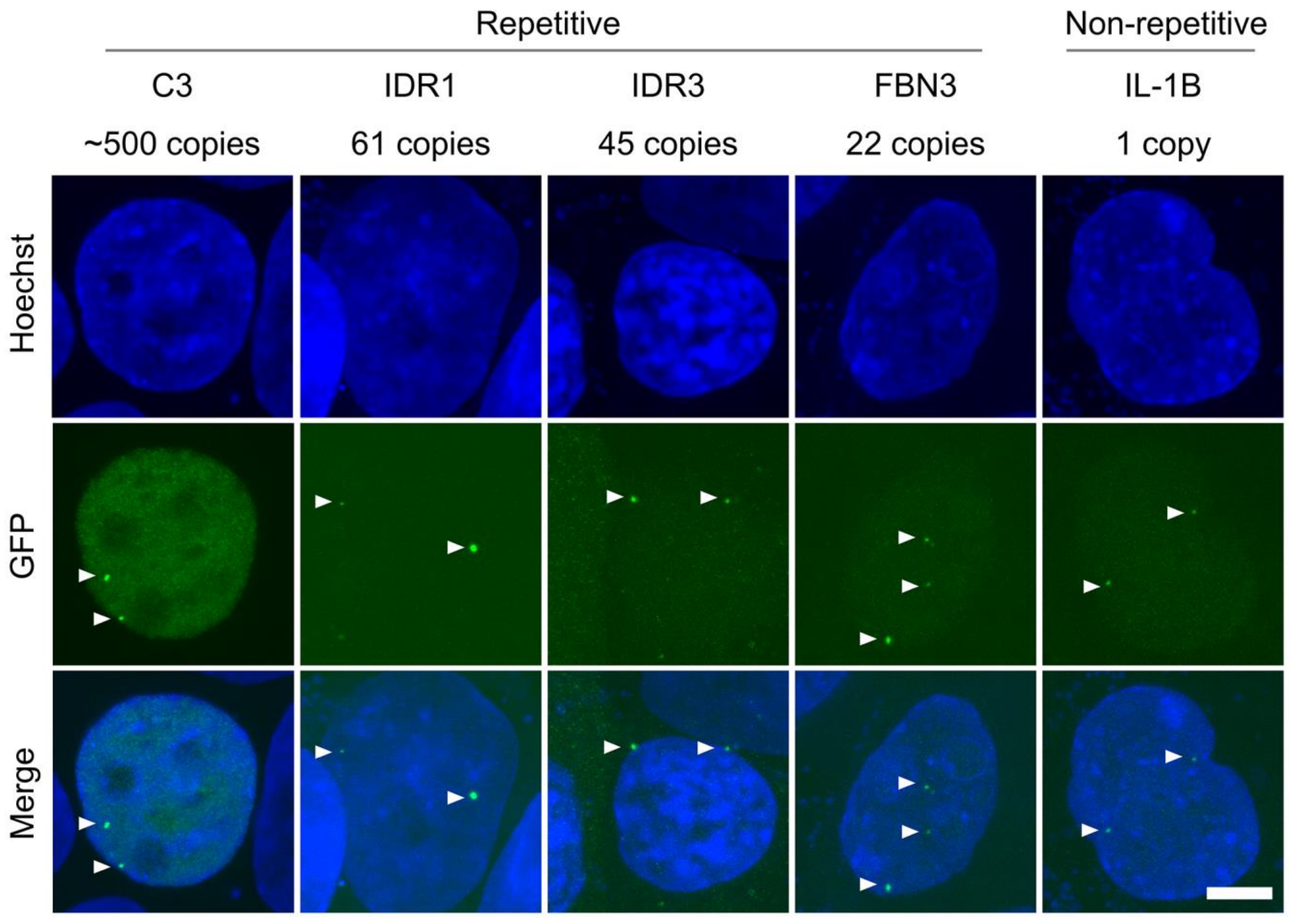


**Figure S11.** Labeling of genomic loci with different copies by split-GFP-coupled CRISPR/Pepper-tDeg. Images are maximum z projections. Scale bar: 5 μm.

**Table S1.** DNA and RNA Sequences used in this work

| Name | Sequences (5’ – 3’) |
|---|---|
| Telomere spacer (Telo) | GUUAGGGUUAGGGUUAGGGUUA |
| Centromere spacer (Centro) | GAAUCUGCAAGUGGAUAUU |
| MUC4.1 spacer | GUACACCCUUGUGUACAGAGCU |
| MUC4.2 spacer | GAAGAGUGGAGGCCGUGCGCGG |
| MUC4.3 spacer | GCAAGCAAGGGAAGCGACAAGG |
| MUC4.4 spacer | GUAAAGUAGAAAAGGCAUAAA |
| C3 spacer | GUGAUAUCACAG |
| IDR1 spacer | GACACCAGUGAC |
| IDR3 spacer | AGCAGAUGUAGG |
| FBN3 spacer | AUCCCUCCAACC |
| IL-1B spacer | UGAGAUAAUUCUCUGGUUCA |
| sgRNA-2×MS2 backbone | GUUUGAGAGCUAGGCCAACAUGAGGAUCACCCAUGUCUGCAGGGCCUAGCAAGUUCAAAUAAGGCUAGUCCGUUAUCAACUUGGCCAACAUGAGGAUCACCCAUGUCUGCAGGGCCAAGUGGCACCGAGUCGGUGC |
| sgRNA-2×Pepper backbone | GUUUGAGAGCUACCGGCUCGUUGAGCUCAUUAGCUCCGAGCCGGUAGCAAGUUCAAAUAAGGCUAGUCCGUUAUCAACUUCCGGCUCGUUGAGCUCAUUAGCUCCGAGCCGGAAGUGGCACCGAGUCGGUGC |
| sgRNA-MS2-Pepper backbone | GUUUGAGAGCUAGGCCAACAUGAGGAUCACCCAUGUCUGCAGGGCCUAGCAAGUUCAAAUAAGGCUAGUCCGUUAUCAACUUCCGGCUCGUUGAGCUCAUUAGCUCCGAGCCGGAAGUGGCACCGAGUCGGUGC |
| F primer of mNeonGreen-tDeg | AAGATCCGGTGGTGGTTCTG |
| R primer of mNeonGreen-tDeg | TCTAGATTAACCGCGGCGAC |
| F primer of circular Pepper | GCCACGTTTCCCACATACTC |
| R primer of circular Pepper | CCGCTTGCCATGAATGATCC |
| F primer of GAPDH | AGAAGGCTGGGGCTCATTTG |
| R primer of GAPDH | AGGGGCCATCCACAGTCTTC |
| TAMRA-Telo | /TAMRA/CCCTAACCCTAACCCTAA |
| Cy5-MUC4 | /Cy5/CTTCCTGTCACCGAC |

**Table S2.** Plasmids used in different experiments

| Assay | Plasmids | Related figures |
|---|---|---|
| Labeling of telomeres by CRISPR/Pepper-tDeg | 0.5 μg of miniCMV-$(mNeonGreen)_4$-tDeg, 0.5 μg of U6-Telo-sgRNA-Pepper | Figure S1 |
| Optimization of FP-tDeg expression level under the control of different promoters | 0.5 μg of miniCMV-$(mNeonGreen)_4$-tDeg or UbC-$(mNeonGreen)_4$-tDeg or EFS-$(mNeonGreen)_4$-tDeg, 0.5 μg of U6-circular Pepper | Figure 1, Figure S2, Figure S4 |
| The proteasomal degradation of mNeonGreen-tDeg | 0.5 μg of miniCMV-$(mNeonGreen)_4$ or 0.5 μg of miniCMV-$(mNeonGreen)_4$-tDeg | Figure S3 |
| Optimization of the nuclear localization of FP-tDeg | 0.5 μg of UbC-$(mNeonGreen)_4$-tDeg or UbC-NLS-$(mNeonGreen)_4$-tDeg, 0.5 μg of U6-circular Pepper | Figure 2a |
| Optimization of the nuclear localization of FP-tDeg | 0.5 μg of UbC-$(mNeonGreen)_4$-tDeg or UbC-NLS-$(mNeonGreen)_4$-tDeg, 0.5 μg of U6-Telo-sgRNA-2×Pepper | Figure 2c, Figure 2d |
| Time-dependent stability of CRISPR/Pepper-tDeg | 0.5 μg of UbC-NLS-$(mNeonGreen)_4$-tDeg, 0.5 μg of U6-Telo-sgRNA-2×Pepper | Figure S5 |
| DNA dual labeling | 0.5 μg of UbC-NLS-$(mNeonGreen)_3$-tDeg, 0.5 μg of UbC-MCP-$(BFP)_3$-NLS, 0.5 μg of U6-Telo-sgRNA-MS2-Pepper | Figure 3b-e, Figure 4a-c |
| DNA dual labeling | 0.5 μg of UbC-MCP-$(BFP)_3$-NLS, 0.5 μg of UbC-NLS-$(mNeonGreen)_3$-tDeg, 0.5 μg of U6-Telo-sgRNA-2×MS2, 0.5 μg of U6-Telo-sgRNA-2×Pepper | Figure S6, Figure S7, Figure S8 |
| DNA dual-color labeling | 0.5 μg of U6-Telo-sgRNA-2×MS2, 0.5 μg of UbC-MCP-$(BFP)_3$-NLS, 0.5 μg of U6-Centro-sgRNA-2×Pepper, 0.5 μg of UbC-NLS-$(mNeonGreen)_3$-tDeg | Figure 3g, Figure 3h, Figure 4d-f |
| Optimization of the number of FPs tagged into tDeg | 0.5 μg of miniCMV-$(mNeonGreen)_4$-tDeg, 0.5 μg of miniCMV-11×$GFP_{11}$-GFP-tDeg-SV40-$GFP_{1\text{-}10}$, 0.5 μg of U6-circular Pepper | Figure S9 |
| Labeling genomic loci with different copies using one sgRNA | 0.5 μg of UbC-NLS-11×$GFP_{11}$-GFP-tDeg-SV40-$GFP_{1\text{-}10}$-NLS, 0.5 μg of U6-Target spacer-sgRNA-2×Pepper (Target spacer = C3, IDR1, IDR3, FBN3, IL-1B, MUC4.1, MUC4.2, MUC4.3, MUC4.4) | Figure 5, Figure S11 |

**Table S3.** Comparison of the proposed system with other CRISPR-based genomic-imaging systems

| **Methods** | **SNR enhancement strategy** | **Labeling sensitivity** | **Real-time tracking** | **Non-repetitive labeling / No. sgRNA** | **Potential issues** | **Ref.** |
|---|---|---|---|---|---|---|
| sgRNA-14×MS2 | Engineered sgRNA-14×MS2 at 3' end for binding FP-fused MCP | Non-repetitive sequences | Yes | Yes / 4 | Potential instability of sgRNA decreasing labeling efficiency | [1] |
| dCas9-SunTag | 24×Suntag amplification system fused into dCas9 | Low-repetitive sequences (≥ 15 copies) | No | No | High "always-on" background signal | [2] |
| CRISPR-Sirus | Engineered sgRNA-Sirius-8×MS2 in stem-loop for binding HaloTag-fused MCP | Low-repetitive sequences (≥ 20 copies) | Yes | No | Decreased sgRNA expression level | [3] |
| BIFC-dCas9/gRNA | BIFC induced by dCas9/gRNA complex and amplified by SunTag system | Low-repetitive sequences (≥ 40 copies) | No | No | Decreased expression level of each component | [4] |
| CRISPR/dual-FRET MB system | Engineered sgRNA to carry two molecular beacons generating FRET | Non-repetitive sequences | Yes | Yes / 3 | Delivery of exogenous nucleic acid probes with sub-optimal retention time | [5] |
| CRISPR LiveFISH | An intrinsic stability switch of fluorescent sgRNA | Repetitive sequences | Yes | No | Labeling sgRNA with fluorophore by sophisticated chemical modification | [6] |
| fCRISPR | Engineered sgRNA-2×Pepper in stem-loop for binding fluorogenic proteins | Low-repetitive sequences (≥ 14 copies) | Yes | No | Insufficient SNR for labeling non-repetitive sequences | [7] |
| CRISPR/ Casilio | Engineered sgRNA at 3' end to carry 15 × Pumilio/FBF (PUF)-binding sites | Non-repetitive sequences | Yes | Yes / 1 | Potential instability of sgRNA to decrease the labeling efficiency;<br>*Nonspecific labeling | [8] |
| CRISPR FISHer | Engineered sgRNA-2×PP7 in stem-loop for binding foldon-GFP-PCP protein trimer | Non-repetitive sequences | Yes | Yes / 1 | Twice transfection required;<br>*Nonspecific labeling | [9] |
| CRISPR SIMBA | Biomolecular assemblies induced by mcherry-HP1α tagged dCas9 through Suntag system. | Non-repetitive sequences | No | Yes / 1 | Rapamycin treatment causing physiological change;<br>*Nonspecific labeling | [10] |
| CRISPR/ Pepper-tDeg | Engineered sgRNA-2×Pepper in stem-loop for binding fluorogenic split-GFP | Non-repetitive sequences | Yes | Yes / 1 | *Nonspecific labeling | This work |

BIFC: Bimolecular fluorescence complementation

*Nonspecific labeling generally happens if fewer sgRNAs are used

**Movie S1.** Live imaging of telomeres in HEK293T cells using CRISPR/MS2-MCP, related to Figure 4a. Scale bar: 5 µm.

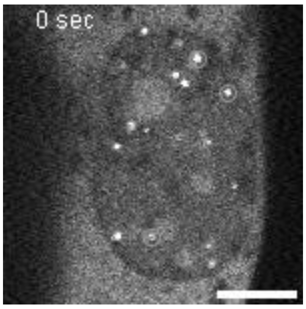


**Movie S2.** Live imaging of telomeres in HEK293T cells using CRISPR/Pepper-tDeg, related to Figure 4a. Scale bar: 5 µm.

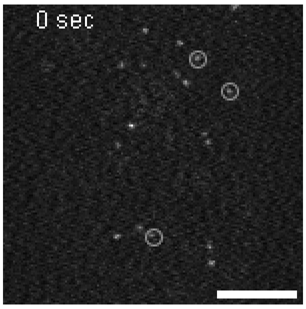

**Movie S3.** Live imaging of telomeres in HEK293T cells using CRISPR/MS2-MCP, related to Figure 4d. Scale bar: 5 μm.

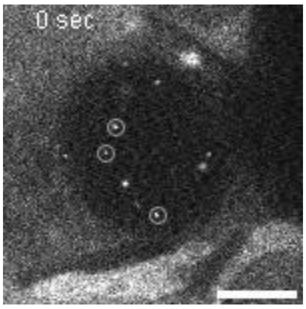


**Movie S4.** Live imaging of centromeres in HEK293T cells using CRISPR/Pepper-tDeg, related to Figure 4d. Scale bar: 5 μm.

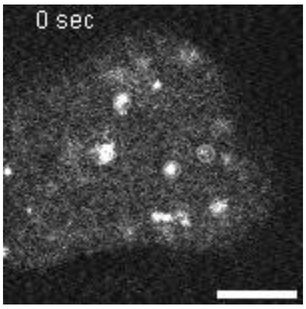

## References

[1] P. Qin, M. Parlak, C. Kuscu, J. Bandaria, M. Mir, K. Szlachta, R. Singh, X. Darzacq, A. Yildiz, M. Adli, *Nat. Commun.* **2017**, *8*, 14725.

[2] H. Ye, Z. Rong, Y. Lin, *Protein Cell* **2017**, *8*, 853-855.

[3] H. Ma, L. C. Tu, A. Naseri, Y. C. Chung, D. Grunwald, S. Zhang, T. Pederson, *Nat. Methods* **2018**, *15*, 928-931.

[4] Y. Hong, G. Lu, J. Duan, W. Liu, Y. Zhang, *Genome Biol.* **2018**, *19*, 39.

[5] S. Mao, Y. Ying, X. Wu, C. J. Krueger, A. K. Chen, *Nucleic Acids Res.* **2019**, *47*, e131.

[6] H. Wang, M. Nakamura, T. R. Abbott, D. H. Zhao, K. W. Luo, C. Yu, C. M. Nguyen, A. Lo, T. P. Daley, M. La Russa, Y. X. Liu, L. S. Qi, *Science* **2019**, *365*, 1301-1305.

[7] Z. Zhang, X. Rong, T. Xie, Z. Li, H. Song, S. Zhen, H. Wang, J. Wu, S. R. Jaffrey, X. Li, *Nat. Commun.* **2024**, *15*, 934.

[8] P. A. Clow, M. Du, N. Jillette, A. Taghbalout, J. J. Zhu, A. W. Cheng, *Nat. Commun.* **2022**, *13*, 1871.

[9] X. Y. Lyu, Y. Deng, X. Y. Huang, Z. Z. Li, G. Q. Fang, D. Yang, F. L. Wang, W. Kang, E. Z. Shen, C. Q. Song, *Cell Res.* **2022**, *32*, 969-981.

[10] Q. Peng, Z. Huang, K. Sun, Y. Liu, C. W. Yoon, R. E. S. Harrison, D. L. Schmitt, L. Zhu, Y. Wu, I. Tasan, H. Zhao, J. Zhang, S. Zhong, S. Chien, Y. Wang, *Nat. Commun.* **2022**, *13*, 7933.
